# Bacterial Regulon Evolution: Distinct Responses and Roles for the Identical OmpR Proteins of *Salmonella* Typhimurium and *Escherichia coli* in the Acid Stress Response

**DOI:** 10.1371/journal.pgen.1004215

**Published:** 2014-03-06

**Authors:** Heather J. Quinn, Andrew D. S. Cameron, Charles J. Dorman

**Affiliations:** 1 Department of Microbiology, Moyne Institute of Preventive Medicine, Trinity College Dublin, Dublin, Ireland; 2 Department of Biology, University of Regina, Regina, Saskatchewan, Canada; Uppsala University, Sweden

## Abstract

The evolution of new gene networks is a primary source of genetic innovation that allows bacteria to explore and exploit new niches, including pathogenic interactions with host organisms. For example, the archetypal DNA binding protein, OmpR, is identical between *Salmonella* Typhimurium serovar Typhimurium and *Escherichia coli*, but regulatory specialization has resulted in different environmental triggers of OmpR expression and largely divergent OmpR regulons. Specifically, *ompR* mRNA and OmpR protein levels are elevated by acid pH in *S.* Typhimurium but not in *E. coli*. This differential expression pattern is due to differences in the promoter regions of the *ompR* genes and the *E. coli ompR* orthologue can be made acid-inducible by introduction of the appropriate sequences from *S.* Typhimurium. The OmpR regulon in *S.* Typhimurium overlaps that of *E. coli* at only 15 genes and includes many horizontally acquired genes (including virulence genes) that *E. coli* does not have. We found that OmpR binds to its genomic targets in higher abundance when the DNA is relaxed, something that occurs in *S.* Typhimurium as a result of acid stress and which is a requirement for optimal expression of its virulence genes. The genomic targets of OmpR do not share a strong nucleotide sequence consensus: we propose that the ability of OmpR to recruit additional genes to its regulon arises from its modest requirements for specificity in its DNA targets with its preference for relaxed DNA allowing it to cooperate with DNA-topology-based allostery to modulate transcription in response to acid stress.

## Author Summary

*Salmonella* Typhimurium is closely related to *Escherichia coli* and they possess identical OmpR DNA binding proteins. *S.* Typhimurium uses OmpR to control the expression of genes involved in adaptation to acid rather than osmotic stress. OmpR expression increases in response to acid stress in *S.* Typhimurium but not in *E. coli* due to structural differences in the *ompR* regulatory region. *S.* Typhimurium OmpR controls many genes, few of which are in *E. coli.* Many OmpR-regulated *S.* Typhimurium-specific targets have been acquired by horizontal gene transfer and contribute to pathogenesis. During infection, *S.* Typhimurium adapts to the macrophage vacuole, an acidic niche where *S.* Typhimurium DNA becomes relaxed. DNA relaxation accompanies acid stress in *S.* Typhimurium but not *E. coli* and enhances OmpR binding to DNA. Drug-induced DNA relaxation mimics the effect of acid stress on OmpR binding to DNA. Thus acid-sensitive OmpR activity in *S.* Typhimurium allows OmpR to control many *S.* Typhimurium-specific genes through a mechanism that depends on changes to DNA topology. We propose that this allosteric role for DNA, combined with a weak requirement on the part of OmpR for binding site sequence specificity, accommodates flexibility in regulon membership and facilitates bacterial evolution.

## Introduction

The relationship between a given regulatory protein and its target genes is subject to evolutionary change, allowing genes to join or to leave a given regulon over time. Evidence for this regulatory flexibility comes from comparisons of orthologous regulators and their regulons from related bacterial species. Four types of variation have been described so far: (i) changes in the presence/absence and the distribution of binding sites for a common regulatory protein at orthologous targets (ii) genes acquired via lateral transfer coming under the control of a regulator already established in the cell (iii) architectural adjustment of a promoter under the control of a regulator to add or subtract regulatory influence and (iv) changes that affect the DNA binding protein itself [1]. The OmpR DNA binding protein of Gram-negative bacteria has the potential to govern collectives of genes whose membership is subject to change. This is because OmpR demonstrates only moderate specificity for its DNA targets [2], allowing new binding sites to arise *de novo* in relatively few mutagenic steps. Furthermore, OmpR has the potential to respond to more than one environmental signal, giving it the potential to participate in regulon evolution from the standpoint of regulatory signal reception. Its activity is under allosteric control through phosphorylation by the EnvZ sensor kinase and the OmpR/EnvZ regulatory cascade is an important component of the osmotic stress response system of *E. coli*. However, OmpR is also sensitive to allosteric effects acting through its DNA target: it binds best to DNA that has adopted a relaxed topology, both *in vivo* and *in vitro* [3] and DNA topology can be modulated by a variety of environmental stressors [4]–[8].

The OmpR/EnvZ two-component system consists of a sensor kinase (EnvZ) located in the cytoplasmic membrane and a response regulator DNA binding protein (OmpR) located in the cytoplasm [9], [10]. The EnvZ protein has two trans-membrane helices, a periplasmic domain and a cytoplasmic domain that undergoes auto-phosphorylation at His-243 [11]. Environmental signal transduction involves phosphorylation of OmpR on Asp-55 by EnvZ [12], [13]. In *E. coli*, this regulatory system has been shown to transmit an osmotic stress signal and among the OmpR targets are the *ompF* and *ompC* genes that encode major porin proteins located in the outer membrane [14]–[16]. Recent evidence shows EnvZ senses changes in osmolarity through its cytoplasmic domain [17].

Mammalian infection by *S.* Typhimurium and *E. coli* exposes the bacteria to acid stress as they transit the stomach. *S*. Typhimurium must also endure acidification within the *Salmonella*-containing vacuole of the host macrophage [18], [19]. In addition to a reduction in pH, the harsh vacuolar environment also exposes the bacterium to Mg^2+^ starvation and toxic defensin peptides [20]. Both species employ general resistance mechanisms that allow them to tolerate external pH values outside the preferred cytoplasmic range. Despite being very closely related at the genetic level, *E. coli* is much more acid-tolerant than *S.* Typhimurium. It can survive pH 2 for several hours whereas *S.* Typhimurium dies rapidly under these conditions [21], [22].

In *S.* Typhimurium, OmpR plays an important role in infection and *ompR* knockout mutants are attenuated for virulence in mice [23]. OmpR regulates the transcription of a number of horizontally acquired genes that are not present in *E. coli* and are key to *S.* Typhimurium virulence [24], [25]. These include the *hilC* and *hilD* regulatory genes in the SPI-1 pathogenicity island [3], a 40-kb genetic element that encodes a type III secretion system and effector proteins required for invasion of mammalian cells [26], [27]. OmpR also regulates the transcription of the *ssrA* and *ssrB* genes of the 40-kb SPI-2 pathogenicity island [3], [28], [29]. These genes encode a two-component system consisting of the sensor kinase (SsrA) and cognate DNA binding protein (SsrB) that regulates the expression of a type III secretion/effector protein system required for intracellular survival and replication of *S.* Typhimurium inside the acidified vacoular compartment [30].

Adaptation to the vacuole involves a complicated process of transcription regulation and this process is modulated by DNA relaxation [31]. Recently, DNA relaxation was shown to enhance the binding of the OmpR protein at specific target genes in SPI-1 (*hilC, hilD*), SPI-2 (*ssrA*) and the core genome (*ompR*) *in vivo* and *in vitro* [3]. Previous work has shown that DNA relaxation occurs in *S.* Typhimurium when the bacterium is exposed to acid stress *in vitro* [4] and that transcription of the *ompR* gene is stimulated by DNA relaxation [3], [32], [33]. These observations led us to hypothesize that exposure of *S*. Typhimurium to low pH might result in elevated levels of active OmpR protein and enhanced binding to its targets throughout the genome. We were also interested to learn if a similar mechanism is used by the closely related bacterium *E. coli* to regulate its OmpR regulon, not least because the two OmpR proteins in these bacterial species have identical amino acid sequences [34, this study]. Our findings show that OmpR expression is regulated differently in *S*. Typhimurium and *E. coli* and that there are very large differences in the composition of the OmpR regulons in these bacterial species. We find that OmpR shows little DNA sequence specificity in its binding sites and propose that this makes OmpR particularly useful for bringing horizontally acquired genes into an established regulatory circuit encoded by the core genome.

## Results and Discussion

### *ompR* gene expression is differentially sensitive to pH in *S.* Typhimurium and *E. coli*

**Figure 1:**
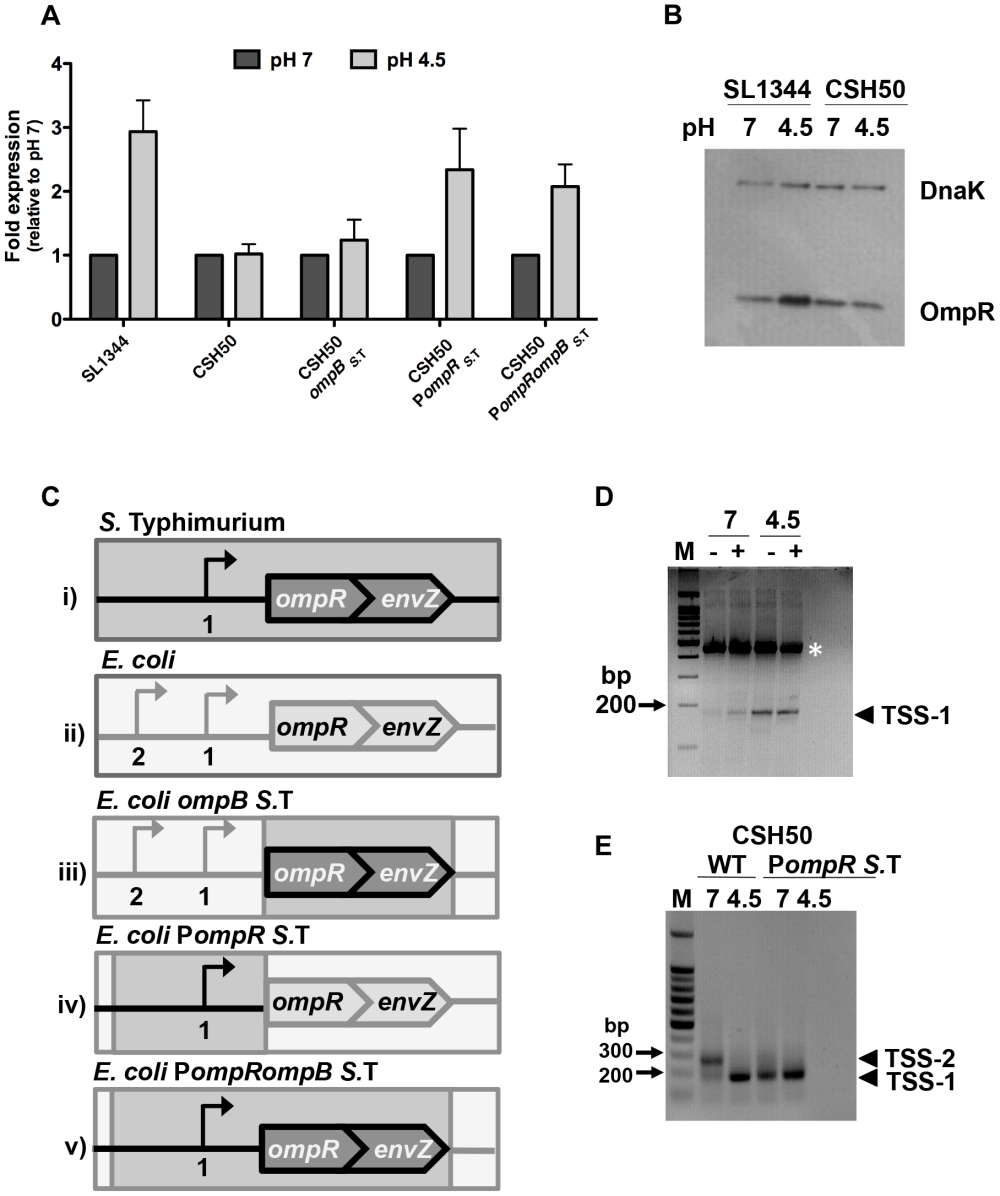
The promoter region is responsible for the pH sensitivity of *ompR* in *S.* Typhimurium. (*A*) Quantitative PCR measurements of *ompR* transcript levels in *S.* Typhimurium (SL1344) and *E. coli* (CSH50) and constructs with exchanged *ompR* regulatory regions (see *C*) at pH 7 and pH 4.5. Mean (N≥3) values are reported and the error bars represent the standard deviation of the mean. (*B*) OmpR protein levels in *S.* Typhimurium (SL1344 *ompR*::3xFLAG) *and E. coli* (CSH50 *ompR*::3xFLAG) at pH 7 and pH 4.5. Anti-FLAG antibody was used to detect the FLAG epitope and DnaK was used as a loading control. (*C*) Diagrams illustrating the constructs (i–v) used in the study. Bent arrows denote transcription start sites (TSSs) The wild type *ompB* locus in *S.* Typhimurium (i). The wild type *ompB* locus in *E. coli* (ii). In *E. coli ompB_S._*_ T._ the native *E. coli ompR* and *envZ* genes are replaced by the corresponding *ompR/envZ* genes from *S.* Typhimurium (dark grey). The native *E. coli ompR* promoter remains (light grey) (iii). In *E. coli* P*ompR _S_*_.T_. the *ompR* promoter in *E. coli* is replaced by the *ompR* promoter from S. Typhimurium (dark grey). The native *E. coli ompB* locus is retained (light grey) (iv). In *E. coli* P*ompR ompB_S_*_. T_. both the *ompR* promoter and *ompR/envZ* genes in *E. coli* are replaced by the *ompR* promoter and *ompR/envZ* genes from *S.* Typhimurium (iv). (*D*) Gel electrophoresis of 5′ RACE-amplified *ompR* cDNA ends analysed on a 3% agarose gel. RNA was extracted after 90 min at pH 7 or pH 4.5. Samples contained either tobacco acid pyrophosphatase (TAP)-treated (+) or untreated (−) RNA, generating 5′ monophosphate for ligation of the RNA-linker (A4; see Table S2) [87]. Ligation of the linker was more efficient in the TAP treated sample. RNA was reverse-transcribed into cDNA and PCR was performed on each cDNA sample using primers RACE_*ompR* and JVO-0367; see Table S2. Arrowhead denotes the PCR product found to be TSS-1. M, 100-bp ladder. The white asterisk denotes a non-specific PCR product that was sequenced and identified as a 23S ribosomal RNA product using BLAST (Basic Local Alignment Search Tool). (*E*) Gel electrophoresis of 5′ RACE-amplified *ompR* cDNA ends analysed on a 1% agarose gel. RNA was extracted from CSH50 and CSH50 P*ompR_S._*_T_ after 90 min at pH 7 or pH 4.5. Arrowheads TSS-1 and TSS-2 denote the PCR products. M, 100-bp ladder. The locations of the transcription start sites are shown in Figure S1.

Because the pH-responsiveness of the *S.* Typhimurium *ompR* promoter does not fit with the *E. coli* osmosensing paradigm, the expression of the orthologous *ompR envZ* operons of *S.* Typhimurium and *E. coli* were compared for pH sensitivity. We used quantitative PCR to measure *ompR* transcript levels in both *S.* Typhimurium (SL1344) and *E. coli* (CSH50) after 90 min at pH 7 or pH 4.5. As expected, *ompR* transcript levels *in S.* Typhimurium were ∼2.7-fold higher at pH 4.5 than at pH 7 (Figure 1A: *S.* Typhimurium). Conversely, the mean level of *ompR* gene expression in *E. coli* was equal at pH 4.5 and at pH 7 (Figure 1A; *E. coli*). OmpR protein levels were measured by western blotting in *S.* Typhimurium strain SL1344 and *E. coli* strain CSH50 that each expressed OmpR with a 3xFLAG epitope fused to its C-terminal domain; the fusion genes were specially engineered to preserve expression and function of the overlapping *envZ* genes. OmpR protein was expressed to a higher level in *S.* Typhimurium at pH 4.5 than at pH 7 whereas its level of expression was constant at pH 4.5 and pH 7 in *E. coli* (Figure 1B). Thus, both *ompR* mRNA and OmpR protein levels were elevated in *S.* Typhimurium in response to acid pH but neither responded to acid pH in *E. coli.* We sought to discover the molecular mechanism responsible for this difference.

### pH sensitivity resides in the *ompB* promoter region

The *ompR* and *envZ* genes constitute the bicistronic *ompB* operon (Figure 1C) [15]. Transcription of *ompB* is driven from the promoter region upstream of *ompR*. An acid-inducible promoter and associated transcription start site (TSS-1) has been identified in this region in *S.* Typhimurium (Figure S1) [33]. Following acid shock, the *ompR* gene is positively autoregulated through a mechanism that requires the sensor kinase EnvZ [33]. We reasoned that species-specific differences in one or more of these factors (*ompB* promoter region, EnvZ protein structure and function) might account for the distinct expression patterns of the *ompR* genes in *S.* Typhimurium and *E. coli* during acid stress.

The nucleotide sequences of the regulatory regions of *ompB* in *S.* Typhimurium and *E. coli* are 88% identical (Figure S1). In addition to differences in nucleotide sequence, the species also differ in the location of *ompR* transcription start sites that have been characterised (Figure S1). The amino acid sequences of the OmpR proteins in the two species are completely identical while those of the EnvZ sensor kinases show a 5% difference in amino acid sequence. Thus the *ompB* regulatory region and the *envZ* gene were analysed as potential contributors to differential expression of *ompR*. This was done by making a series of transgenic strains in which the regulatory regions and the *ompR envZ* open reading frames from *S.* Typhimurium were exchanged individually and then together for their *E. coli* counterparts (Figure 1C). Quantitative PCR was used to measure *ompR* transcript levels after 90 min growth at pH 7 or pH 4.5.

*E. coli ompB_S._*_T_ has had the open reading frames of its own copies of the *ompR* and *envZ* genes replaced by the corresponding genes from *S.* Typhimurium; it retains the *E. coli ompB* promoter region. Transcription of *ompR* in this strain was similar at both pH 4.5 and 7, i.e. it retained the previously observed *E. coli* pattern of *ompR* expression (Figure 1A; *E. coli ompB_S._*_T_) implicating the promoter region in conferring pH sensitivity. *E. coli* P*ompR_S._*_ T_. has had the promoter region of its *ompB* operon replaced with the equivalent region from *S.* Typhimurium; it retains its *E. coli ompR* and *envZ* open reading frames. In this strain the *ompR* gene showed enhanced expression at pH 4.5 (Figure 1A; *E. coli* P*ompR_S._*_ T_.), showing that it has acquired the *S.* Typhimurium pattern of *ompR* gene expression in response to acid stress. In *E. coli* P*ompR_ S._*_ T_. *ompB_S._*_ T._ the entire *S.* Typhimurium *ompB* locus and its regulatory region has replaced the equivalent region of the *E. coli* genome. This strain expresses *ompR* in the acid-inducible pattern that is characteristic of *S.* Typhimurium (Figure 1A; *E. coli* P*ompR_S._*_ T._ *ompB_ S._*_ T._). Transcription of the *envZ* gene, which is 3′ to *ompR* in the bicistronic *ompB* operon, showed a similar pH response to that of *ompR* (Figure S2A). EnvZ protein levels did not show a marked response to acid pH, either in SL1344 itself or when the *S.* Typhimurium *ompB* operon and its promoter were transferred to the *E. coli* chromosome (Figure S2B) indicating that while *envZ* transcription shares the pH sensitivity of *ompR*, its posttranscriptional response to pH is distinct.

These data showed that the *S.* Typhimurium *ompR* promoter region determines the low-pH sensitivity that is a characteristic of the *S.* Typhimurium *ompR* gene. Further, pH sensitivity can be conferred upon *E. coli* even in the presence of endogenous *E. coli* EnvZ, which is tuned to osmotic sensing. Thus the 5% difference in amino acid sequence between the *E. coli* and *S.* Typhimurium EnvZ proteins is not the basis of the differential sensitivity of *E. coli* and *S.* Typhimurium *ompR* expression to pH.

### The *ompR* regulatory region has pH-sensitive transcription start sites in both species

Previous studies have mapped *ompR* transcription start sites in both *S.* Typhimurium and *E. coli* [33], [35]–[38]. In this study we focussed on *ompR* acid-inducible TSSs, examining the possibility that *S.* Typhimurium and *E. coli* each utilises a unique *ompR* TSS profile at different pH values thus explaining the pH sensitivity of *ompR* in *S.* Typhimurium. The transcription architectures of the *E. coli* and *S.* Typhimurium *ompR* genes were compared at pH 4.5 and pH 7 using 5′ RACE (Figure 1D, E). The previously described acid-inducible transcription start site (TSS-1) in *S.* Typhimurium was confirmed; the transcript initiating at this position was detectable at both pH values but was more abundant at acidic pH (Figure 1D). In *E. coli* strain CSH50 two transcripts were detected at pH 7, with one being much more abundant than the other; this pattern of predominance was reversed at pH 4.5 (Figure 1E). The predominant transcript at pH 7 (henceforth called TSS-2) mapped to the same location as that identified in *E. coli* by Tsui *et al.* [36]. The second and previously unidentified *E. coli* transcript (TSS-1) was present at pH 7 and pH 4.5. We mapped TSS-1 in *E. coli* to a location that was identical to that of TSS-1 in *S.* Typhimurium. Therefore, although overall *ompR* transcript levels in *E. coli* appear to be pH insensitive (Figure 1A) there is a clear shift in TSS utilisation and the output of TSS-1-originating transcription in both species under acid conditions (Figure 1D, E). We next mapped TSSs in the *E. coli* derivative harbouring the *S.* Typhimurium *ompR* regulatory region (Figure 1E; *E. coli* P*ompR_S._*_ T._). We found that the *S.* Typhimurium *ompR* promoter maintained its native transcriptional start site profile in an *E. coli* background and that only a single transcript from TSS-1 was detected at both pH values. Thus, in response to changes in pH, a re-prioritising of TSS usage occurs in *E. coli*. This results in a distinct species-specific TSS profile at *ompR* and directly influences the level of OmpR protein produced in either species (Figure 1B). We next wished to investigate what effect this differential response to pH may have on the OmpR regulon in both organisms.

### Genome-wide binding of OmpR in *S.* Typhimurium and *E. coli*

We investigated the impact of acid pH on genome-wide binding of OmpR to assess the physiological consequences of differential *ompR* gene regulation in each species. Chromatin immunoprecipitation-on-chip (ChIP-on-chip) was used to ascertain the *in vivo* distribution of OmpR on the chromosomes of *S.* Typhimurium and *E. coli* at pH 4.5 and pH 7 using *S.* Typhimurium strain SL1344 *ompR*::3xFLAG and *E. coli* strain CSH50 *ompR*::3xFLAG, respectively. We have previously shown the OmpR protein with a FLAG tag at the C-terminus is functional [3]. To generate a ‘snapshot’ of OmpR binding, DNA-protein interactions were cross-linked after 90 min at pH 4.5 or pH 7 and OmpR-protein-DNA complexes were immunoprecipitated using an anti-FLAG antibody. As a control for possible noise produced by non-specific immunoprecipitation during ChIP-chip, we performed a control ‘mock’ ChIP-chip experiments under identical culture conditions. We used normal mouse IgG antibody during the immunoprecipitation step, the log2 ratio was then subtracted from the experimental log2 ratio. The 100% identity of the amino acid sequences of the OmpR proteins in the two species allowed direct comparisons to be made of OmpR binding patterns in these two organisms. In considering our data we kept in mind the fact that binding of OmpR to a particular gene was not in itself evidence that OmpR regulates the expression of that gene.

**Figure 2:**
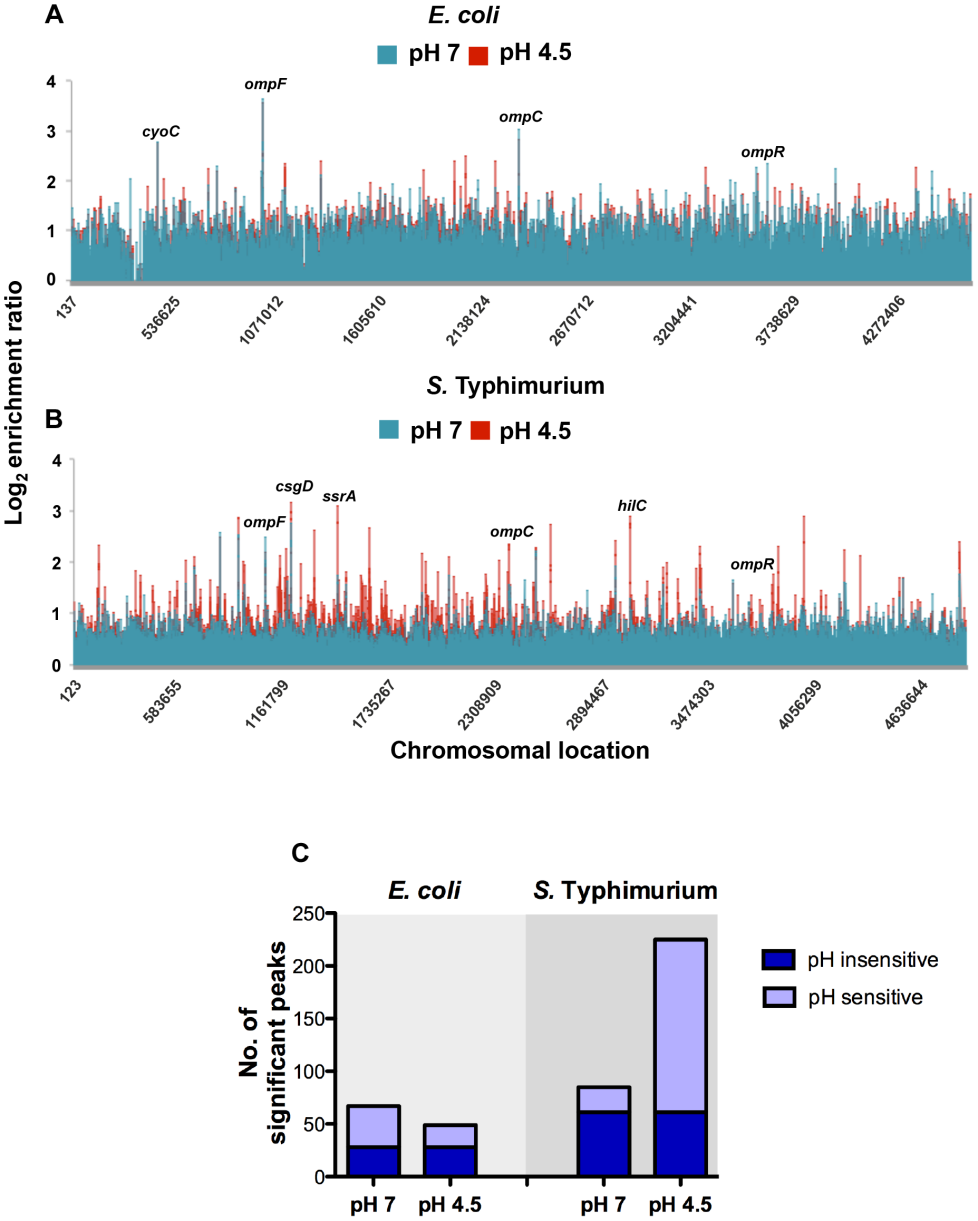
Genome-wide distribution of OmpR in *E. coli* and *S.* Typhimurium. Results from genome-wide analysis of OmpR binding in *E. coli* (*A*) and *S.* Typhimurium (*B*) at pH 7 (blue) and pH 4.5 (red). The log_2_ enrichment ratio (ChIP/input) is plotted on the *y*-axis and the locations of the probes are shown on the *x*-axis. (*C*) Overlap between OmpR binding sites at pH 7 and pH 4.5 in *E. coli* and *S.* Typhimurium. The histogram illustrates the number of significant OmpR binding peaks bound at pH 7 and pH 4.5 in *E. coli* and *S.* Typhimurium. The number of targets occupied at pH 7 and pH 4.5 is shown in dark blue (pH sensitive). The number of pH-specific targets occupied at pH 7 or pH 4.5 is shown in light blue (pH insensitive).

OmpR binding was increased at low pH throughout the chromosome of *S.* Typhimurium as indicated by a larger number of DNA binding peaks in the pH 4.5 samples compared to the corresponding samples from *E. coli* (Figure 2A, B). The ChIPOTle ChIP data analysis program was used to identify high-confidence OmpR binding regions in each species. In *S.* Typhimurium, ChIPOTle identified 85 significantly enriched OmpR binding regions at pH 7 and this increased to 225 peaks at pH 4.5 (Figure 2C). Of these OmpR-bound regions, 61 were detected in both pH 7 and pH 4.5 conditions (Figure 2C; pH insensitive). In *E. coli*, ChIPOTle analysis identified 67 OmpR binding regions at pH 7, and 49 binding regions at pH 4.5; 28 OmpR-targets were bound at both pH 7 and pH 4.5 (Figure 2C; pH insensitive). The results showed that OmpR bound more targets in *S.* Typhimurium at acidic pH compared to neutral pH (Figure 2C) whereas in *E. coli* there was a small decrease in the number of targets bound by OmpR at acidic pH. Perhaps the greater abundance of OmpR at pH 4.5 in *S.* Typhimurium compared to *E. coli* (Figure 1C) accounts, at least in part, for these differences in protein binding patterns. We detected binding of OmpR to previously identified OmpR-regulated genes, validating our experimental approach. These genes were: *ompF*, *ompC* [34], [37]–[42], *tppB* [43], [44], *csgD* [45]–[47], *ompR* [32], [33], *flhD* [48], *ssrA* [3], [28], [29], *hilC* (3] *omrA* [49], *omrB* [49], *hyaB2* [49] and *hyaA2* [50].

In addition to previously characterised OmpR targets, we found OmpR bound at genes associated with pH homeostasis in both organisms. In *E. coli* OmpR bound the *cadBA* operon (Figure S5C) that encodes an acid stress response system responsible for consuming intracellular protons by decarboxylation of a specific amino acid substrate [51]. In *S.* Typhimurium OmpR was bound at the *atpI* gene encoding a component of the F_1_F_o_ ATP synthase. This enzyme promotes ATP-dependent extrusion of protons and ATP synthesis under acidic conditions [52]. We also identified OmpR binding at additional ATPases (e.g. STM1635, STM0723 and *ybiT*) and to cation transporters (e.g. *ybaL*, STM0765 and STM3116). OmpR binding to acid stress genes in both species indicates that OmpR performs a shared function of regulating pH homeostasis. Surprisingly, we found that overall there was only a small number of common OmpR targets between these organisms.

### The *S.* Typhimurium and *E. coli* OmpR regulons have limited overlap

Only 15 OmpR targets were found to be in common between both organisms (Table S3), indicating significant divergence in the *E. coli* and *S.* Typhimurium OmpR regulons. In both species, OmpR binds its own promoter, as well as the well characterised *E. coli* targets *ompF* and *ompC*. In the context of differences in the OmpR regulons of *E. coli* and *Salmonella*, it is interesting to note that *ompC* expression is differentially affected by osmolarity in *E. coli* and *S.* Typhi [34]. Although OmpR had very few common target genes in *S.* Typhimurium and *E. coli*, those it did bind shared the common theme of contributing to cell envelope composition. These include the small RNA gene *rseX* which downregulates the expression of RNAs encoding the outer membrane porins OmpC and OmpA [53]. Other cross-species OmpR targets included factors involved in biofilm formation, motility, chemotaxis and genes involved in fimbrial production such as *csgD*, the regulator of curli synthesis and assembly [45], [46]. OmpR bound the *flhDC* operon that encodes the master flagellar regulator in both species. OmpR has been shown to repress expression of *flhDC* in *E. coli* [54], [48]. We noted OmpR binding was elevated at the *lrhA-alaA* intergenic region. *lrhA* encodes the LysR-like protein LrhA which is a global regulator of flagellar, motility and chemotaxis genes [55] including *flhDC.* LrhA also regulates expression of type 1 fimbriae that promote attachment to host cells and biofilm formation on abiotic surfaces [56]. OmpR binding was also increased at the *ycb* operon (an orthologue of *bcfA* in *S.* Typhimurium) that encodes cryptic fimbriae for attachment to abiotic surfaces [57].

Interestingly, the pattern of OmpR binding was not conserved at all members within the shared regulon. For example OmpR binding at pH 4.5 was increased at *micF* in *S.* Typhimurium but not in *E. coli* (Figure S3A). OmpR binding was similar and pH insensitive at the *ompF* gene in both species (Figure S2, B). OmpR binding at *rseX* was more abundant at pH 4.5 in *E. coli* but not in *S.* Typhimurium (Figure S3C) suggesting that although total OmpR protein levels in *E. coli* are insensitive to pH OmpR levels at target genes are influenced by pH. These results suggest that common gene targets may differ in the detail of their OmpR mediated regulation between *S.* Typhimurium and *E. coli*. The core OmpR regulon includes genes encoding surface expressed appendages such as outer membrane porins, curli fibres, motility factors and fimbriae. These targets are consistent with a generalized role for OmpR in the regulation of cell surface composition in both organisms. The retention of these targets in both species is not surprising because the bacterial envelope is the first barrier against acidic pH and OmpR is known to regulate expression of the outer membrane porins in response to shifts in external pH such that expression of the smaller-channel porin OmpC is favoured over OmpF [58]. Following from this any species-specific OmpR targets absorbed into the OmpR regulon may represent examples of regulon evolution enabling niche adaptation.

### *S.* Typhimurium-specific OmpR targets and DNA topology

During infection orally-ingested *S.* Typhimurium experience acidic pH in both the host stomach and within the acidified-*Salmonella*-containing vacuole of the host macrophage [18]–[20]. Acidic pH is both a threat to *S.* Typhimurium's survival and an environmental cue directing *S.* Typhimurium to upregulate expression of virulence genes [59]. We found OmpR targeted some of these virulence genes located within the horizontally acquired pathogenicity islands -1 and -2 (SPI-1 and SPI-2) and that binding there was pH sensitive. SPI-1 and -2 are required for infection and enable *S.* Typhimurium to invade host epithelium and survival inside host macrophage respectively [24]. Crucially, these islands are absent from *E. coli* [25], thus these OmpR targets were acquired by the OmpR regulon after lateral DNA transfer. In keeping with findings from previous studies we found OmpR binding to be increased within SPI-1 and SPI-2 [3], [28], [29]. In addition we found OmpR bound at SPI effector genes located outside these islands providing further evidence of regulon evolution (discussed below).

**Figure 3:**
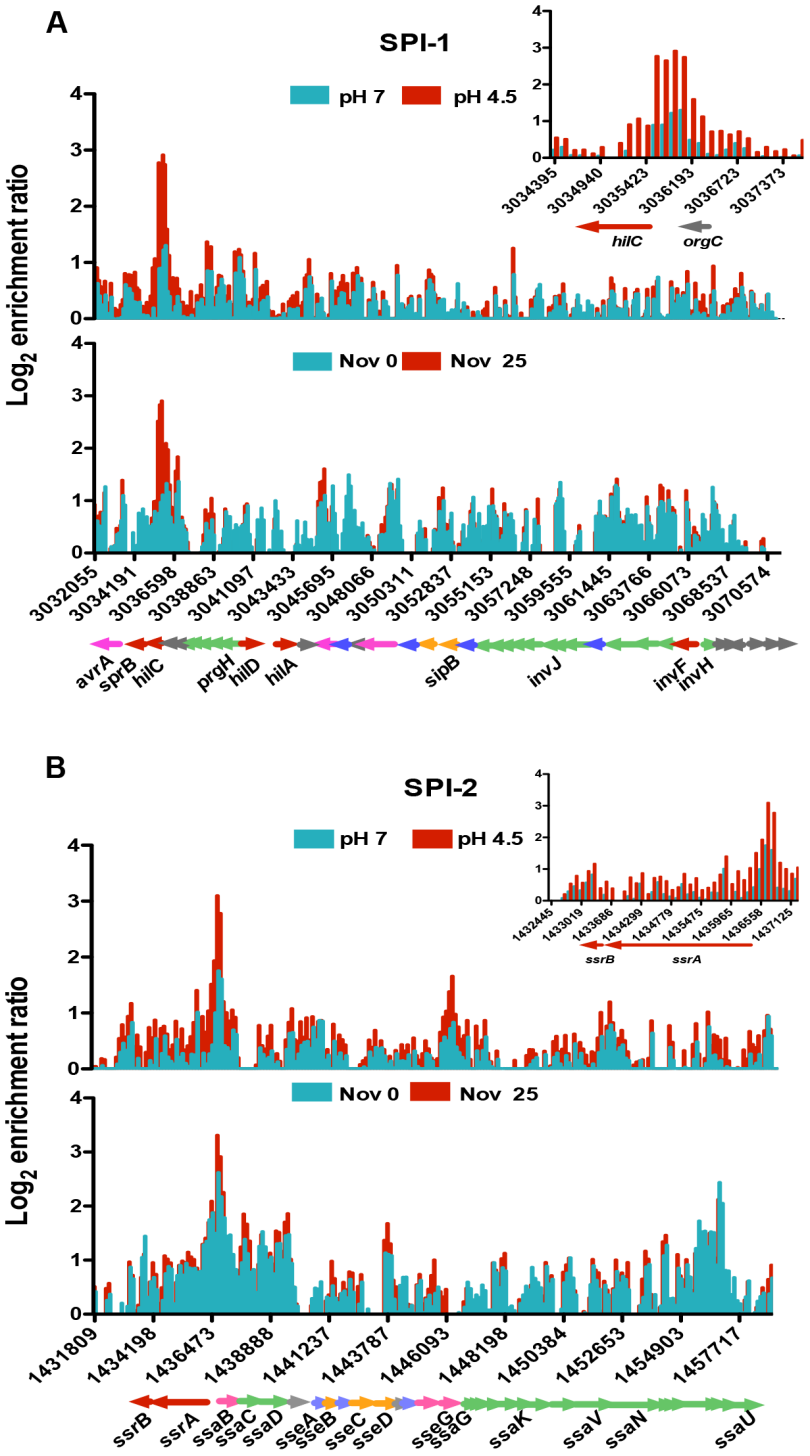
The effect of pH and DNA relaxation on OmpR binding across *Salmonella* pathogenicity islands 1 and 2 (SPI-1 and SPI-2). (*A*) Log_2_ enrichment ratio (ChIP/input) is plotted on the *y*-axis and the locations of the probes are shown on the *x*-axis for SPI-1. Coloured arrows below the *x*-axis show the locations of SPI-1 genes determined using Jbrowse [38]. The inset shows the OmpR binding pattern at the *hilC* regulatory gene of SPI-1 in more detail. (*B*) Log_2_ enrichment ratio (ChIP/input) is plotted on the *y*-axis and location of the probes are shown on the *x*-axis for SPI-2. Genes colour is based on their function regulatory genes are red, effector genes are pink, structural genes are green, translocon genes are orange, chaperone genes are blue and genes of unknown function are grey. The inset shows the OmpR binding pattern at the *ssrA ssrB* regulatory genes of SPI-2 in more detail. In (*A*) and (*B*), the top histogram shows OmpR binding at pH 7 (blue) and pH 4.5 (red) and the bottom histogram shows OmpR binding without (blue) and with (red) novobiocin treatment (25 µg ml^−1^). Genes are coloured to indicate their function: regulatory genes are red, effector genes are pink, structural genes are green, translocon genes are orange, chaperone genes are blue and genes of unknown function are grey.

SPI-1 expression is triggered by multiple environmental signals and is regulated by the SPI-1-encoded HilC and HilD proteins. Together these activate expression of HilA and in turn this activates expression of SPI-1 structural genes encoding a type III secretion system (T3SS) [60], [61]. Expression of the SPI-1 genes also requires core-genome-encoded regulators such as OmpR, Fis and H-NS [62]–[65]. OmpR acts as a positive regulator of *hilC* and a negative regulator of *hilD* expression [3]. Our ChIP analysis revealed OmpR binding to be significantly increased upstream of the *hilC* regulatory gene (Figure 3A, inset) and the level of this binding was higher still in acidic conditions. In addition, OmpR binding was elevated in the *prgH-hilD* intergenic region and within the *prgHIJK* operon which encodes structural components of the SPI-1 needle complex that delivers bacterial effectors to host cell [66].

SPI-2 gene expression is dependent on the SsrA/SsrB two-component system; where the SsrB response regulator activates SPI-2 structural genes encoding a T3SS [67], [68]. OmpR binds to the *ssrA* promoter and positively regulates its transcription [3], [28], [29]. We found that within SPI-2, OmpR was most significantly enriched in the intergenic region of the divergently transcribed *ssrA* and *ssaB* genes. OmpR binding there increased at low pH (Figure 3B, inset) coinciding with the 6-fold OmpR-dependent increase in *ssrA* transcript levels detected at pH 4.5 (Figure S4). OmpR also bound the *sseA* promoter that controls expression of the genes within the effector/chaperone operon of SPI-2. Binding was also detected within the coding sequence of *sseG* and *ssaV* the latter of which forms an essential structural component of the T3SS. [69]. The effect of OmpR binding within the coding sequences of these genes is unknown.

We found OmpR bound at both SPI-1 and -2 secreted effector protein genes located outside these pathogenicity islands. These included the SPI-1 effector genes *sopE2*, *sopA* and *gtgE* and the SPI-2 effector genes *sseK2 gogB*, *pipB2*, *sopD2* and *gtgE* [20]. Thus, the relationship between OmpR and the pathogenicity islands is complex and OmpR is likely to regulate virulence in *S.* Typhimurium at levels beyond its influence at the SPI master regulators *ssrA/ssrB*, *hilC* and *hilD*. In keeping with this possibility, we detected elevated OmpR binding in the laterally-acquired pathogenicity island SPI-4 (Figure S5A) that encodes a type 1 secretion system for transporting the SiiE large adhesin to the cell surface (Figure S5B) [70]. This non-fimbrial adhesin is required for attachment and invasion of polarized epithelial cells during the intestinal phase of infection [71]. OmpR binds directly to SPI-4 within the coding region of the *siiABCDE* genes; an effect on gene expression here would provide a further example of the expansion and evolution of the OmpR regulon to control pathogenicity gene expression.

Whilst we observed an increase in OmpR protein levels in *S.* Typhimurium at low pH we also considered the possibility that DNA topology influenced OmpR binding to its chromosomal targets. Two lines of evidence supported the hypothesis that OmpR binding to its DNA targets in acid-stressed cells is modulated by the topology of the target DNA. First, acid treatment of *S.* Typhimurium results in relaxation of plasmid DNA supercoiling [4]. Second, we found that OmpR binding to the *hilC, hilD, ompR*, and *ssrA* promoters was enhanced by relaxation of DNA supercoiling [3].

**Figure 4:**
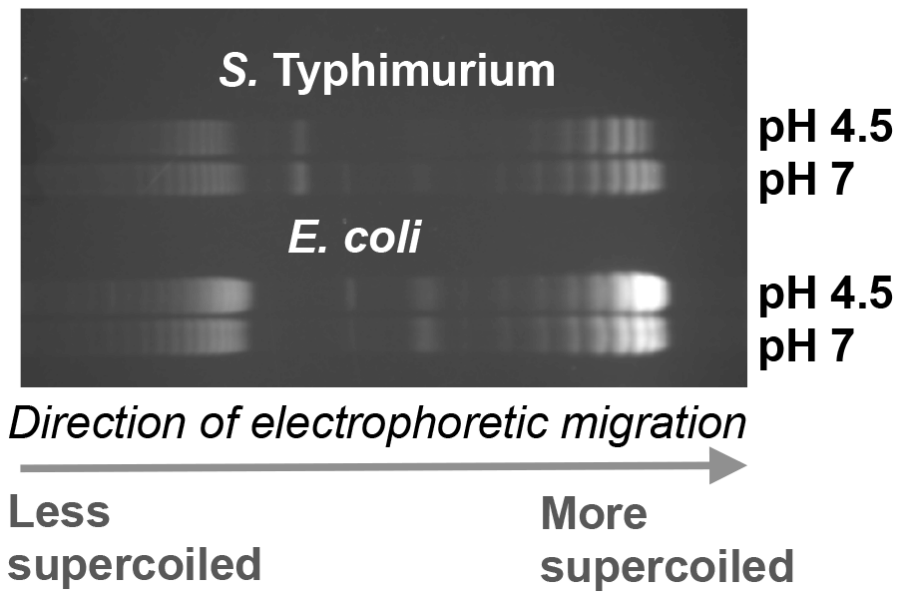
The effect of pH on DNA supercoiling in *Salmonella* Typhimurium strain SL1344 (top) and *E. coli* strain CSH50 (bottom). Electrophoretic mobility of plasmid pUC18 topoisomers in agarose gel containing chloroquine at 2.5 µg ml^−1^. At this concentration of chloroquine more supercoiled topoisomers run further in the gel. Cultures were pelleted after 90 min at pH 7 or pH 4.5 E-minimal medium and plasmids were extracted immediately. Gel image is representative of three independent experiments.

We investigated the possibility that global relaxation of DNA supercoiling caused by acidic pH contributes to the dramatic increase in OmpR regulon size at pH 4.5 in *S.* Typhimurium. First we measured DNA supercoiling in *S.* Typhimurium after 90 min at pH 7 or pH 4.5 and found that reporter plasmid DNA became more relaxed at acid pH (Figure 4) in agreement with previous findings [4]. Interestingly, we found DNA in *E. coli* became more supercoiled at low pH, this is quite clear in the plasmid dimers (Figure 4). This is important as we have previously shown DNA in both species can be artificially relaxed using the drug novobiocin [72]. Despite this, we find acidic pH relaxes DNA topology in *S.* Typhimurium but not in *E. coli*.

We mapped OmpR binding using ChIP-on-chip in *S.* Typhimurium cells treated with a sub-inhibitory concentration of the DNA-gyrase-inhibiting drug novobiocin during exponential growth. DNA becomes relaxed in these cells because novobiocin blocks access to the ATP binding pocket of the GyrB subunit of DNA gyrase, preventing that copy of the gyrase enzyme from performing a further round of negative DNA supercoiling [73]. Strikingly, the OmpR ChIP-on-chip profiles at acidic pH and those obtained after novobiocin treatment showed similar increases in OmpR binding at several loci (Figure 3A, B); OmpR binding was focused at the *hilC* promoter in SPI-1 and at the *ssrA* promoter within SPI-2. Thus, acidic pH and relaxation of DNA supercoiling due to DNA gyrase inhibition created very similar OmpR binding profiles across the major pathogenicity islands of *S.* Typhimurium.

### OmpR and the PhoQ/PhoP regulon of *S.* Typhimurium

**Figure 5:**
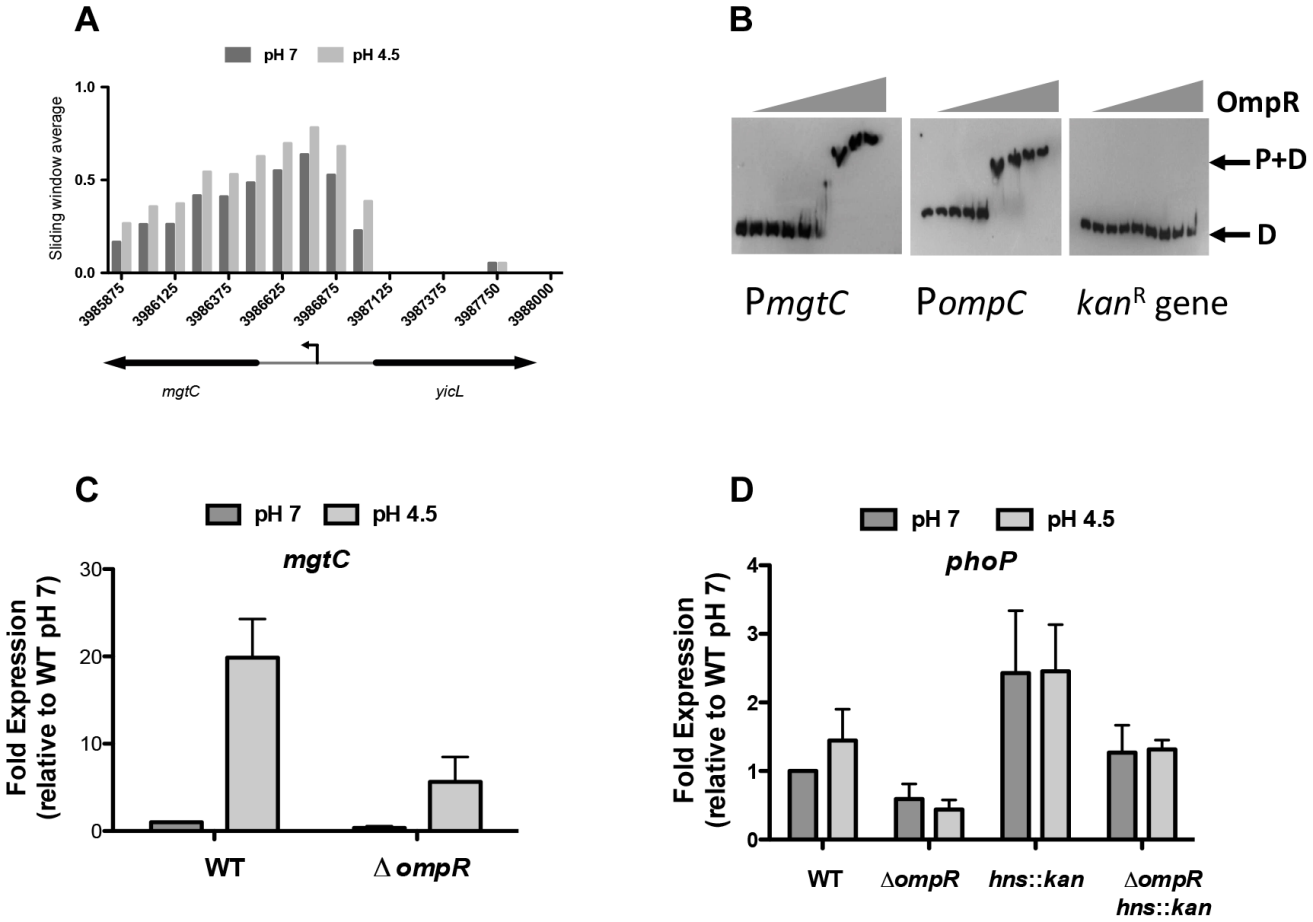
OmpR regulates PhoP-regulated genes. (*A*) OmpR binding at *mgtC* at pH 7 and pH 4.5. Sliding window average of log2 enrichment as calculated by ChIPOTle [88] is shown on the *y-*axis. (*B*) EMSA analysis showing OmpR binding to the *mgtC* promoter as well as the *ompC* promoter (positive control) and the *kan* ^R^ (*kanamycin* resistance gene; negative control). D, free DNA probe; P+D, protein + DNA complex. OmpR concentrations used were 0, 0.02, 0.04,.078,.16, 0.31, 0.63, 1.25, 2.5 µM. A representative gel image is shown from three independent replicates (*C*) Quantitative PCR measurements of *mgtC* transcript levels at pH 7 and pH 4.5 in the wild type strain and an *ompR* knockout mutant. (*D*) Quantitative PCR measurements of *phoP* transcript levels at pH 7 and pH 4.5 in the wild type strain and an *ompR* knockout mutant and in the *ompR hns*::*kan* double mutant. N≥3; standard deviations from the mean are shown as error bars.

At low pH, OmpR binding was elevated at several genes that belong to the PhoQ/PhoP two-component system regulon (Table S4). In *S.* Typhimurium, the PhoP DNA binding protein regulates genes involved in virulence, magnesium transport, intramacrophage survival and resistance to antimicrobial peptides, with PhoP activity being dependent on phosphorylation by the membrane-associated sensor kinase PhoQ [74], [75]. Our ChIP-chip data showed increased binding of OmpR at the promoter region of *mgtC*, a PhoP-regulated gene that encodes an inner membrane protein involved in intramacrophage survival (Figure 5A) [76]. Electrophoretic mobility shift analysis (EMSA) confirmed that OmpR binds specifically to the *mgtC* promoter (Figure 5B). A DNA probe encompassing the *ompC* promoter which is a well-characterized OmpR target was included as a positive control. A DNA probe encompassing a portion of the kanamycin resistance gene which contains no known OmpR binding sites was included as a negative control. OmpR bound the *ompC* promoter and did not bind the kanamycin gene as expected (Figure 5B). Using qRT-PCR we found that *mgtC* expression was induced at pH 4.5 and required OmpR (Figure 5C). Thus, OmpR is an important activator of *mgtC* expression.

OmpR binding was also elevated at low pH at the PhoP-activated genes *pagK* and *pagO* and at the PhoP-repressed operon *prgHIJK*, as indicated earlier in the discussion of SPI-1. OmpR also targeted the PhoP-dependent *mig14* gene that encodes a protein mediating resistance to the cathelicidin-related antimicrobial peptide CRAMP [77] (Table S4.).

In addition to overlap with the PhoP regulon, OmpR was found to bind and regulate the *phoP* promoter. Genetic evidence indicated that OmpR was required for fine-tuning *phoP* expression both in the presence and absence of H-NS (Figure 5D). However, ChIP data indicated that the level of OmpR binding at *phoP* was not significantly above that seen in the mock immunoprecipitated control. A potential OmpR binding site that was identified bioinformatically at the *phoP* promoter was mutated by site-directed mutagenesis (Figure S6). The result was a mild reduction in the ability of OmpR to bind here; approximately 50% of the wild-type P*phoP* DNA shifted at 2 µM OmpR whereas >2 µM OmpR protein was required to shift ∼50% of the mutant OmpR-I^-^ P*phoP*. These results suggest that OmpR positively regulates *phoP* via a mechanism that remains to be elucidated.

The PhoQ/PhoP and EnvZ/OmpR two-component systems are found throughout the *Enterobacteriaceae* and the results presented here demonstrate an overlap between their regulons in *S.* Typhimurium. The genes listed in Table S4 are absent from *E. coli*, with the exception of *slyB* and *phoP*. The *slyB* and *phoP* genes are PhoP-regulated in *E. coli*, yet we did not detect OmpR binding at these genes. This observation indicates that the integration of the OmpR and PhoP regulatory circuits may have occurred after divergence of *S.* Typhimurium and *E. coli*. Many of the *Salmonella* genes in Table S4 are associated with virulence and their expression is governed by PhoP either directly or indirectly, this may enable these shared targets respond to multiple signals sensed by either the OmpR/EnvZ and PhoP/PhoQ two-component systems.

### OmpR binding site motif analysis

**Figure 6:**
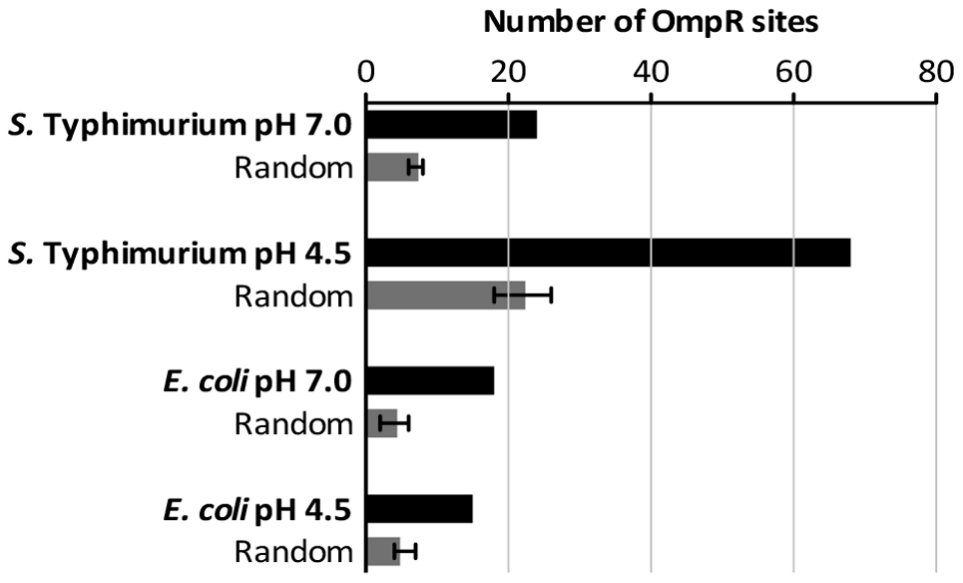
The number of high-scoring OmpR sites identified within the ChIP datasets. Sequences of the binding sites are listed in Table S6. Random DNA datasets were generated as described in *Materials and Methods*.

OmpR demonstrates only moderate sequence specificity for binding sites in DNA [2]; consequently, the biochemically-characterized OmpR binding sites in Regulon DB do not reveal a good consensus for the binding site [78]. Using MEME we conducted an unbiased motif search of the DNA sequences from our ChIP-chip datasets that were bound by OmpR [79]. We tested for the presence of any sequence motif that may be enriched in DNA bound by OmpR, but no significant motif was detected in the *E. coli* or *S*. Typhimurium ChIP datasets. We next conducted a biased search using a position weight matrix built from characterized *E. coli* and *S.* Typhimurium OmpR binding sites (listed in Table S5) that contain elements of the GTnTCA motif to which OmpR binds with high affinity [2]. Searches were performed using the RSAT matrix-scan program [80], and putative OmpR sites were identified in all our ChIP-chip datasets (listed in Table S6). To control for random matches to the weight matrix, we analysed in parallel replicate datasets composed of random DNA sequence generated by the RSAT random-sequence program and matched to the size of the experimental ChIP datasets. In all cases, the OmpR-bound sequences from the ChIP-chip datasets contained significantly more putative OmpR sites than the random DNA sequences (Figure 6) consistent with the presence of specific OmpR binding sites in the experimental ChIP datasets. Perhaps it is unsurprising that no OmpR logo was identified in our ChIP datasets by unbiased motif searching given the low DNA sequence-specificity displayed by the OmpR protein for binding. This may also explain the observation of numerous weak peaks of OmpR binding detected throughout the genomes of both *S.* Typhimurium and *E. coli* (Figure 2A, B). The lack of an OmpR binding site motif and genome-wide binding may allow OmpR to act as a global regulator of transcription and to incorporate newly-acquired genes into its regulon, as observed with SPI-1 and SPI-2. OmpR binding sites that contain the GTnTCA motif may represent a subclass of high-specificity OmpR targets that nucleate formation of higher-order nucleoprotein structures.

### Perspective

**Figure 7:**
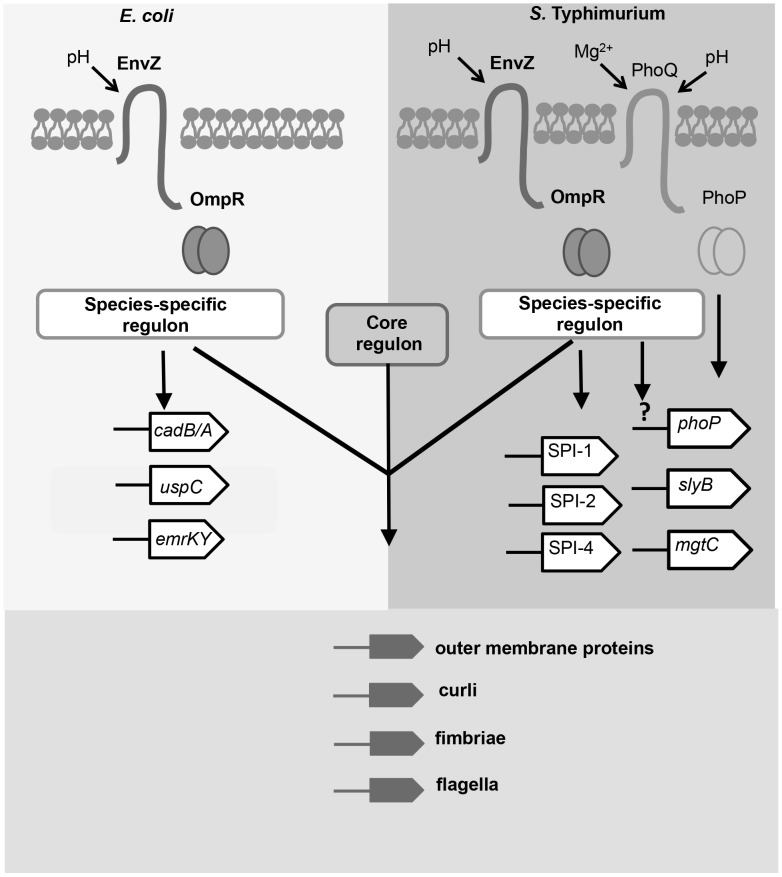
The OmpR regulons of *S.* Typhimurium and *E. coli* in acid pH. Members of the OmpR regulon in has evolved since the divergence of these closely-related species. OmpR (pairs of circles) binds to a species-specific regulon in *Salmonella* and *E. coli*; examples of these targets are shown. In *E. coli*, OmpR binds to genes involved in acid resistance e.g. *cad* operon [13] and general stress resistance e.g. the *uspC* [14] and *emrK* [15] genes. In *S.* Typhimurium, OmpR binds to pathogenicity islands SPI-1, -2, and -4, and to genes regulated by PhoP. OmpR positively regulates *phoP* expression by an unknown mechanism (denoted by the question mark). The genes identified as the core OmpR regulon (conserved targets; bound by OmpR in both species) encode surface-associated organelles and proteins. Modulation of the cell-surface composition may be an important function of the core OmpR regulon in response to acidic stress.

The OmpR protein in *S.* Typhimurium binds to a much larger number of targets than does its identical orthologue in *E. coli*. It bound to the same gene in the two species in only 15 cases; these genes make up the core OmpR regulon and all contribute in some fashion to the composition of the cell surface. We found members of the species-specific regulons included genes involved in pathogenesis in *S.* Typhimurium and stress-management in *E. coli* (Figure 7). Although OmpR levels did not change in acid-treated *E. coli*, OmpR did appear to assume a pH-specific role in that species. For example, OmpR targeted the genes of the lysine/cadaverine decarboxylase system that is a part of the acid stress response system [51]. Such a pH-specific role for OmpR in *E.* coli is in agreement with recent findings that identified OmpR as a regulator of the acid stress response in *E. coli* [81].

The large OmpR regulon in *S.* Typhimurium includes many genes that have been acquired by horizontal gene transfer. In many cases, the same genes are targets of H-NS-mediated transcription silencing and OmpR is known to be an effective antagonist of H-NS silencing when accompanied by DNA relaxation [3]. OmpR and H-NS share a weak requirement for specific sequences for DNA binding, although in both cases high-affinity consensus sequences have been described [41], [64], [82]. Thus, these proteins are especially suitable for imposing dual control at promoters that have at least the DNA structural features if not the specific sites that these proteins require for binding. In the case of OmpR, relaxed DNA is a better target for binding than is supercoiled DNA, as has been shown *in vitro* and *in vivo* [3; this work]. In this context it is interesting to note that the *mgtC* gene is a target for acid-pH-dependent positive regulation by OmpR (Figure 5) and it encodes a protein that inhibits the activity of the F_1_F_o_ ATP synthase [75]. DNA gyrase requires ATP to supercoil DNA negatively so a reduction in ATP results in a general relaxation of cellular DNA [7], [8], [73], [83]. These observations suggest that a regulatory circuit exists in which the production of ATP is down-regulated in *S.* Typhimurium in low-pH growth conditions, resulting in up-regulation of OmpR expression and a concomitant enhancement of OmpR binding to its genomic targets, which is reinforced by the negative impact of MgtC on ATP synthesis. The operation of such a circuit is consistent with empirical data showing that DNA in *S.* Typhimurium becomes relaxed when the bacterium is in the low-pH environment of the macrophage vacuole [31].

Our findings demonstrate an allosteric role for DNA topology in the operation of the OmpR regulon in *S.* Typhimurium. OmpR interacts with DNA via both a helix-turn-helix (H-T-H) motif and a winged helix [2]. The H-T-H motif interacts with the major groove in DNA while the wing contacts the minor groove [2]. A+T-rich DNA sequences have a narrow minor groove whose width is adjustable by changes in DNA supercoiling [84]. This structural variation provides a mechanism for modulating the interaction of OmpR with a given DNA target that is additional to any allosteric control that operates at the level of the protein. Our finding that OmpR binds to many A+T-rich genes that have been acquired by HGT in *S.* Typhimurium is particularly interesting given that its interactions with those targets shows the greatest sensitivity to DNA topological change. The H-NS nucleoid-associated protein targets these genes too and silences their transcription, but H-NS binding seems to less affected by relaxation of the DNA target than is OmpR [3]. These two proteins seem to be particularly well matched for the purpose of imposing environmentally-responsive dual control on A+T-rich genes: H-NS binds and silences them while OmpR binds and antagonises H-NS-mediated repression but only when both the OmpR protein and its DNA target are appropriately primed by allosteric control. Newly acquired genes that meet the structural requirements for this type of dual control may be expected to make good candidates for membership of the OmpR regulon, contributing to the evolution of the bacterium.

## Materials and Methods

### Strains and growth media

*Salmonella* Typhimurium serovar Typhimurium strain SL1344, *Escherichia coli* K-12 strain CSH50 and isogenic derivatives used in this study are listed in Table S1. Cells were grown in E-minimal medium [85] supplemented with L-histidine (0.5 mM), L-proline (100 µg ml^−1^) and thiamine (1 µg ml^−1^) or LB broth at 37°C and 200 rpm. Antibiotics were used at the following final concentrations; chloramphenicol 20 µg ml^−1^; kanamycin 50 µg ml^−1^; carbenicillin 100 µg ml^−1^.

### Culture conditions

To measure the effect of pH on *ompR* expression and OmpR binding cells were grown overnight in LB broth. The culture optical density (OD_600_) was equalized to 0.15 units. Cells were collected by centrifugation and washed with 1 ml EG-minimal medium pH 7. This was repeated twice more to remove residual nutrients from the LB broth. Cells were grown in 40 ml EG-minimal medium (pH 7) in a 250 ml flask to an OD_600_ ∼0.5–0.6 at 37°C and 200 rpm. The culture was then split into two 20 ml volumes and collected by centrifugation at room temperature. One cell pellet was resuspended in 1 ml EG-minimal medium at pH 7, the second pellet was resuspended in 1 ml EG-minimal medium at pH 4.5. Each suspension was added to a final volume of 20 ml pre-warmed EG-minimal medium of the same pH in a 250 ml flask and kept at 37°C and 200 rpm for 90 min before harvesting for analysis. To measure the effect of novobiocin treatment cells were treated as previously described [3]. Novobiocin was used at a final concentration of 25 µg ml^−1^ and cells treated for 40 min before harvesting for ChIP analysis.

### Cloning and mutant construction

Detailed information on cloning and mutant construction can be found in S1 text.

### Western blotting

Cells were normalized to 0.2 OD_600_ units and western blot analysis was performed as previously described [3]. Anti-FLAG (F3165, Sigma-Aldrich) was used at 1 in 10,000 and Anti-DnaK (1.0 mg ml^−1^, Enzo life sciences) was used at 1 in 200,000. Goat anti-mouse IgG horseradish peroxidase conjugate (Millipore) was used at 1 in 10,000. Autoradiography was used to visualize chemiluminescent emissions derived from horseradish peroxidase oxidation of luminol, blots were developed using Hyperfilm MP (Amersham Biosciences) film.

### Electrophoretic mobility shift assays

These were performed using purified OmpR (D55E) protein as previously described [3] with the following exceptions; biotinylated primers were used to PCR amplify DNA probes (*mgtC*; P*mgtC*_F_EM and P*mgtC*_R_EM, *ompC*; P*ompC_*EM_F and P*ompC_*EM_R, *kan* gene; *kan*_bio_F and *kan*_bio_R, *phoP*; Bio_P*phoP*_F and Bio_P*phoP*_R listed in Table S2). After running the gels as described previously the gels were transferred to a Biodyne B membrane (Pall) for 1 h at 30 V in 0.5 X TBE buffer. Blots were UV cross-linked (GS GeneLinker, Bio-Rad) and developed using the Chemiluminescent nucleic acid detection module (Thermoscientific).

### RNA extraction, cDNA synthesis and quantitative PCR

Total RNA was extracted from SL1344 and CSH50 cells normalized to 2.0 OD_600_ units. Cultures were prepared as previously described [38]. RNA was then extracted using the SV Total RNA Isolation System (Promega) according to manufacturers instructions. 50 µl of RNA was DNase treated using the Turbo-DNA-free kit (Ambion). RNA was analyzed on a 1% TAE agarose gel. 1 µg of RNA was synthesized to cDNA using the GoScript Reverse Transcription System (Promega) according to manufacturers instructions. 100 µl nuclease-free H_2_O was added to each cDNA pool. Quantitative PCR was performed using oligonucleotides listed in Table S2. Primers specific for *ompR* in SL1344 were *ompR*_RT_S.e_F and *ompR*_RT_S.e_R, *ompR* in CSH50; *ompR*_RT_E.c_F and *ompR*_RT_E.c_R, *ssrA* in SL134; *ssrA*_RT_F and *ssrA*_RT_R, *mgtC* in SL1344 *mgtC*_RT_F and *mgtC*_RT_R, *phoP* in SL134; *phoP_*S.e_RT_F and *phoP* S.e_RT_R. PCR reactions were performed as previously described [3] Primers specific for the *gmk* gene were used as an internal control as previously described [86], SL1344 specific *gmk* primers are listed *gmk*_F_S.e and *gmk*_R_S.e and CSH50 specific *gmk* primers are listed *gmk*_F_E.c and *gmk*_R_E.c in Table S2.

### 5′RACE

This was performed as previously described [87] using RNA extracted from SL1344 and CSH50 after 90 min in EG-minimal medium pH 7 or pH 4.5. PCR was then performed on cDNA samples using adapter-specific (JVO-0367; see Table S2) and *ompR* specific primer (RACE_*ompR*; see Table S2). The oligonucleotide RACE_*ompR* is complementary to a region of conserved nucleotide sequence in both species. PCR products were cloned into the linearized vector pJET (Fermentas) and transformed into *E. coli* strain XL-1 and at least 5 clones were DNA sequenced.

### Motif analysis

Unbiased motif finding was conducted using MEME 4.9, and the following parameters were tested: motifs could range in size from 10 to 50 bp, each DNA sequence could contain multiple or no motif sites, and motifs could be palindromic or non-palindromic. The RSAT matrix-scan program was run with default settings except that the background model was based on the genome subset of non-coding upstream DNA from *E. coli* K12 or *S*. Typhimurium LT2, depending on the ChIP dataset being analyzed. The RSAT random-sequence program used the nucleotide hexamer frequencies in non-coding upstream sequences from *E. coli* K12 or *S*. Typhimurium LT2 to generate random sequence datasets of sizes matching the experimental datasets.

### Plasmid topoisomer gel electrophoresis

The high-copy plasmid pUC18 was used to report DNA supercoiling levels in this study [7]. Cultures were harvested after 90 min at pH 7 and pH 4.5 and plasmid DNA was extracted using the Promega PureYield plasmid miniprep system. Plasmid DNA was ran on an agarose gel containing 2.5 µg ml^−1^ chloroquine with 2X Tris Borate EDTA (TBE) used as gel and running buffer as previously described [3].

### Chromatin immunoprecipitation and microarray analysis

ChIP was performed as previously described [64] using the strains SL1344 *ompR*::3xFLAG and CSH50 *ompR*::3xFLAG. Anti-FLAG antibody (Sigma-Aldrich, cat no. F3165) was used to immunoprecipitated the OmpR-FLAG tagged protein. Normal mouse IgG (Millipore) was used as a control for non-specific (‘mock’) immunoprecipitation. Fluorescent labelling of DNA was performed as previously described [64]. Samples were prepared for hybridisation as follows: 50 µl 2X Hi-RPM Hybridisation buffer (Agilent), 25 µl ChIP or ‘mock’ DNA, 5 µl input DNA, 10 µl 10X GE blocking Agent (Agilent) and 10 µl nuclease-free H_2_O. Samples were added to a microfuge tube, vortex-mixed briefly and collected by short centrifugation spin. This was then added to the appropriate species-specific (SL1344 or MG1655) microarray slide (Oxford Gene Technology; 4×44 K identical arrays). Arrays were sealed with the appropriate gasket slide, loaded into the Agilent Microarray Hybridisation Chamber Kit (G2534A) and hybridised for 24 h at 65°C in a hybridization oven (Agilent). Slides were washed according to instructions provided by Oxford Gene Technology and scanned on Agilent High-Resolution C scanner at 635 and 532 nm. The median intensities for both channels were acquired by Agilent Feature Extraction Software version 10.5.1.1. This software calculates the background fluorescence for each spot. These values were used to calculate the background subtracted ChIP/input ratio. The data was median-normalised by calculating the median fluorescence for each channel (Cy3 or Cy5) and using a scaling factor to ensure the median of the data set was equal to 1. Three independent ChIP-on-chip replicates were performed for SL1344 and CSH50 experiments in EG-minimal medium at pH 7 and pH 4.5. Two independent ChIP-on-chip replicates were performed for SL1344 experiments without novobiocin and with novobiocin at 25 µg ml^−1^. Two independent control ChIP-on-chip replicates were performed using ‘mock’ antibody in SL1344 and CSH50 backgrounds. The median ChIP/input ratio and the mock/input ratio were calculated. The median log_2_ ratio for each independent biological replicate was calculated. To correct for non-specifically enriched peaks, the mock median log_2_ ratio was subtracted from each ChIP median log_2_ ratio. The log_2_ ratios were used in the ChIPOTle programme [88] to define peaks of enrichment as described previously [64] a *P*-value of *P* = 0.001 was assigned. The complete ChIP-on-chip datasets have been submitted to the Gene Expression Omnibus (GEO) database (accession number GSE49914).

## Supporting Information

Figure S1 Nucleotide sequence alignment of the *ompR* regulatory regions of *S.* Typhimurium and *E. coli*. Alignment of the *ompR* regulatory region of *S. enterica* Typhimurium (Top) and *Escherichia coli* (Bottom) is shown. Conserved nucleotides are indicated by asterisks (*). Transcription start sites (TSS) are highlighted in grey and in bold in the appropriate sequence. TSS-1 [33] and TSS-2 [36] have been characterized previously. The ATG start codon is in bold and italicized. The −10 and −35 motifs for the *E. coli* TSS-1 and *S. enterica* TSS-1 are underlined. The CAT start codon for the divergently transcribed *greB* gene is in bold.
(TIF)

Figure S2 The effect of pH on *envZ* transcript and EnvZ protein levels. (*A*) Quantitative PCR measurements of *envZ* transcript levels in *S.* Typhimurium (SL1344) and *E. coli* (CSH50) and constructs with exchanged *ompR* regulatory regions (see Figure 1C) at pH 7 and pH 4.5. Mean (N≥3) values are reported and the error bars represent the standard deviation of the mean. (*B*) EnvZ protein levels in *S.* Typhimurium (SL1344 *envZ*::3xFLAG) *and E. coli* (CSH50 *envZ*::3xFLAG) at pH 7 and pH 4.5. Anti-FLAG antibody was used to detect the FLAG epitope and DnaK was used as a loading control.
(TIF)

Figure S3 OmpR binding at genes in the core OmpR regulon. OmpR binding at pH 7 and pH 4.5 for *ompC* (*A*) *ompF* (*B*) and *rseX* (*C*) in *E. coli* (left panel) and *S.* Typhimurium (right panel). Arrows below indicate location and orientation of open reading frames. Bent arrows show small RNAs. Sliding window average of log2 enrichment as calculated by ChIPOTle [88] is shown on the *y-*axis. The bell-shaped curve shown here arises from DNA fragments with an OmpR binding sites located close to their centre hybridizing more frequently to the microarray than those with the binding site located toward the DNA ends.
(TIF)

Figure S4 OmpR activates *ssrA* expression at pH 7 and pH 4.5. Quantitative PCR measurements of *ssrA* transcript levels at pH 7 and pH 4.5 in WT and the Δ*ompR* mutant. N≥3; standard deviations of the mean are shown as error bars.
(TIF)

Figure S5 Increased OmpR binding at *Salmonella* pathogenicity island −4 and the *cadBA* operon in *E. coli*. Increased OmpR binding at pH 7 and pH 4.5 within SPI-4. (*A*) OmpR binding within SPI-4 as measured at pH 7 and pH 4.5. Arrows denote open reading frames and their orientation. Sliding window average of log2 enrichment as calculated by ChIPOTle [88] is shown on the *y-*axis. (*B*) Illustration of the SPI-4 type 1 secretion system (T1SS) and secretion of the SiiE adhesin (black curved line). SiiF is an inner membrane transporter ATPase, SiiD is a periplasmic adaptor protein and SiiC is an outer membrane protein. The functions of the SiiA and SiiC proteins are unclear; they may be interaction partners that regulate the retention of SiiE (adapted from 70). (*C*) Increased OmpR binding within the *cadB*-*cadC* intergenic region. Arrows denote open reading frames and their orientation. Sliding window average of log2 enrichment as calculated by ChIPOTle [88] is shown on the *y-*axis.
(TIF)

Figure S6 Electrophoretic mobility shift assay of OmpR binding to the *phoP* promoter. EMSA analysis showing OmpR binding to the wild-type *phoP* promoter (WT) and the *phoP* promoter harbouring the mutated (i.e. OmpR-I^-^) binding site. D, free DNA probe; P+D, protein + DNA complex. OmpR concentrations used were: 0, 0.5, 1, 2, 4, 8, and 16 µM.
(TIF)

Table S1 Strains and plasmids used in this study. The table provides details of the strains of *Escherichia coli*, *Salmonella enterica* serovar Typhimurium and plasmids used in the experiments described in the text. The sources of these materials or references to papers giving this information is also included.
(DOCX)

Table S2 Oligonucleotides used in this study. The table reports the DNA sequences of primers used for cloning, quantitative PCR, mutant construction, DNase I footprinting or electrophoretic mobility shift assays (bandshifts).
(DOCX)

Table S3 Common OmpR targets in SL1344 and CSH50. The table lists those genes that are common to *S.* Typhimurium and *E. coli* and that were bound by OmpR protein in ChIP-chip experiments.
(DOCX)

Table S4 PhoP-regulated genes identified as OmpR targets. The table lists the genes that are known to be regulated by the PhoP protein in *S.* Typhimurium and that were bound by the OmpR protein in the ChIP-chip experiments.
(DOCX)

Table S5 List of OmpR sites used to build OmpR weight matrix. The table lists the DNA sequences of those OmpR binding sites that were used to construct the weight matrix for OmpR.
(DOCX)

Table S6 List of OmpR sites found in the ChIP-on-chip data sets for SL1344 and CSH50 at pH 7 and pH 4.5. The table lists the genes in *S.* Typhimurium and *E. coli* that were bound by the OmpR protein in the ChIP-chip experiments.
(DOCX)

Text S1 This text provides details of the construction of mutant bacterial strains in which components of the *ompB* locus from *S.* Typhimurium were transferred to *E. coli*, the production of genes expressing FLAG-tagged OmpR and EnvZ proteins and a description of the site-directed mutagenesis of the regulatory region of the *phoP* gene in *S.* Typhimurium. Relevant references are also included.
(DOCX)
