# Supplementary figures and images for: Bacterial Regulon Evolution: Distinct Responses and Roles for the Identical OmpR Proteins of Salmonella Typhimurium and Escherichia coli in the Acid Stress Response
Source: PLoS Genet. 2014 Mar 6;10(3):e1004215. doi: 10.1371/journal.pgen.1004215 (PMC3945435; doi:10.1371/journal.pgen.1004215)

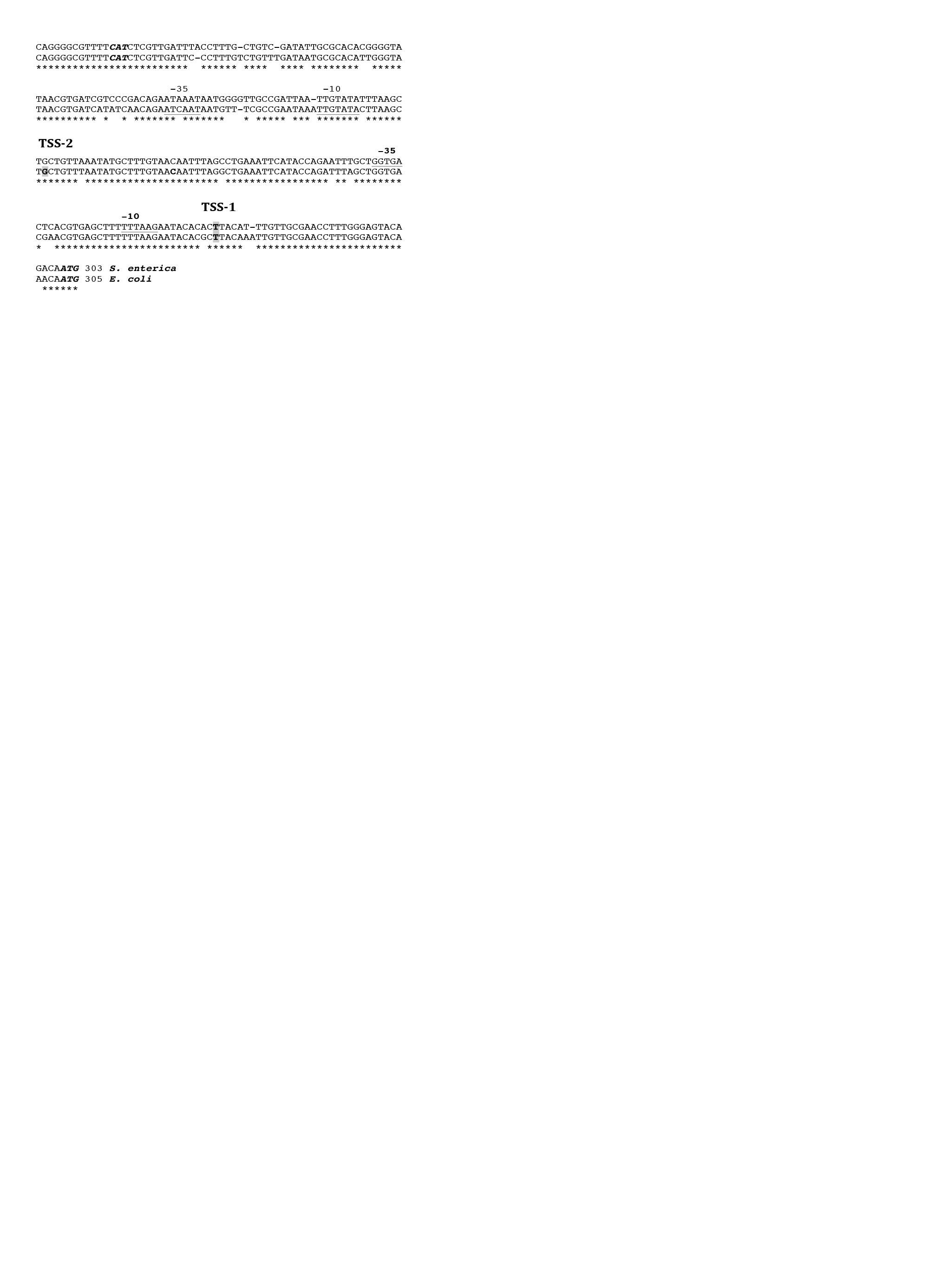

Supplement: Figure S1 — Nucleotide sequence alignment of the ompR regulatory regions of S. Typhimurium and E. coli. Alignment of the ompR regulatory region of S. enterica Typhimurium (Top) and Escherichia coli (Bottom) is shown. Conserved nucleotides are indicated by asterisks (*). Transcription start sites (TSS) are highlighted in grey and in bold in the appropriate sequence. TSS-1 [33] and TSS-2 [36] have been characterized previously. The ATG start codon is in bold and italicized. The −10 and −35 motifs for the E. coli TSS-1 and S. enterica TSS-1 are underlined. The CAT start codon for the divergently transcribed greB gene is in bold. (TIF) [file pgen.1004215.s001.tif]

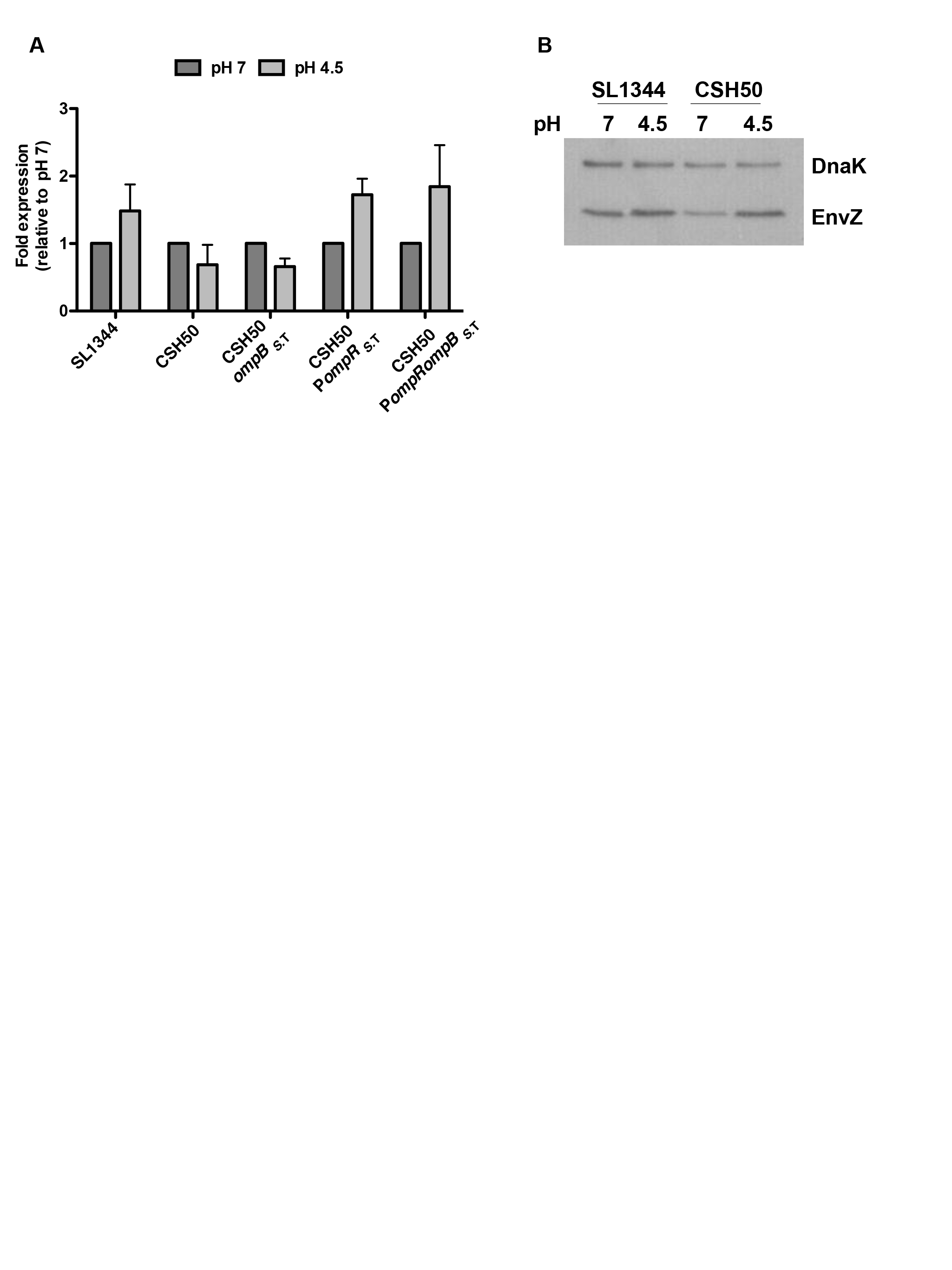

Supplement: Figure S2 — The effect of pH on envZ transcript and EnvZ protein levels. (A) Quantitative PCR measurements of envZ transcript levels in S. Typhimurium (SL1344) and E. coli (CSH50) and constructs with exchanged ompR regulatory regions (see Figure 1C) at pH 7 and pH 4.5. Mean (N≥3) values are reported and the error bars represent the standard deviation of the mean. (B) EnvZ protein levels in S. Typhimurium (SL1344 envZ::3xFLAG) and E. coli (CSH50 envZ::3xFLAG) at pH 7 and pH 4.5. Anti-FLAG antibody was used to detect the FLAG epitope and DnaK was used as a loading control. (TIF) [file pgen.1004215.s002.tif]

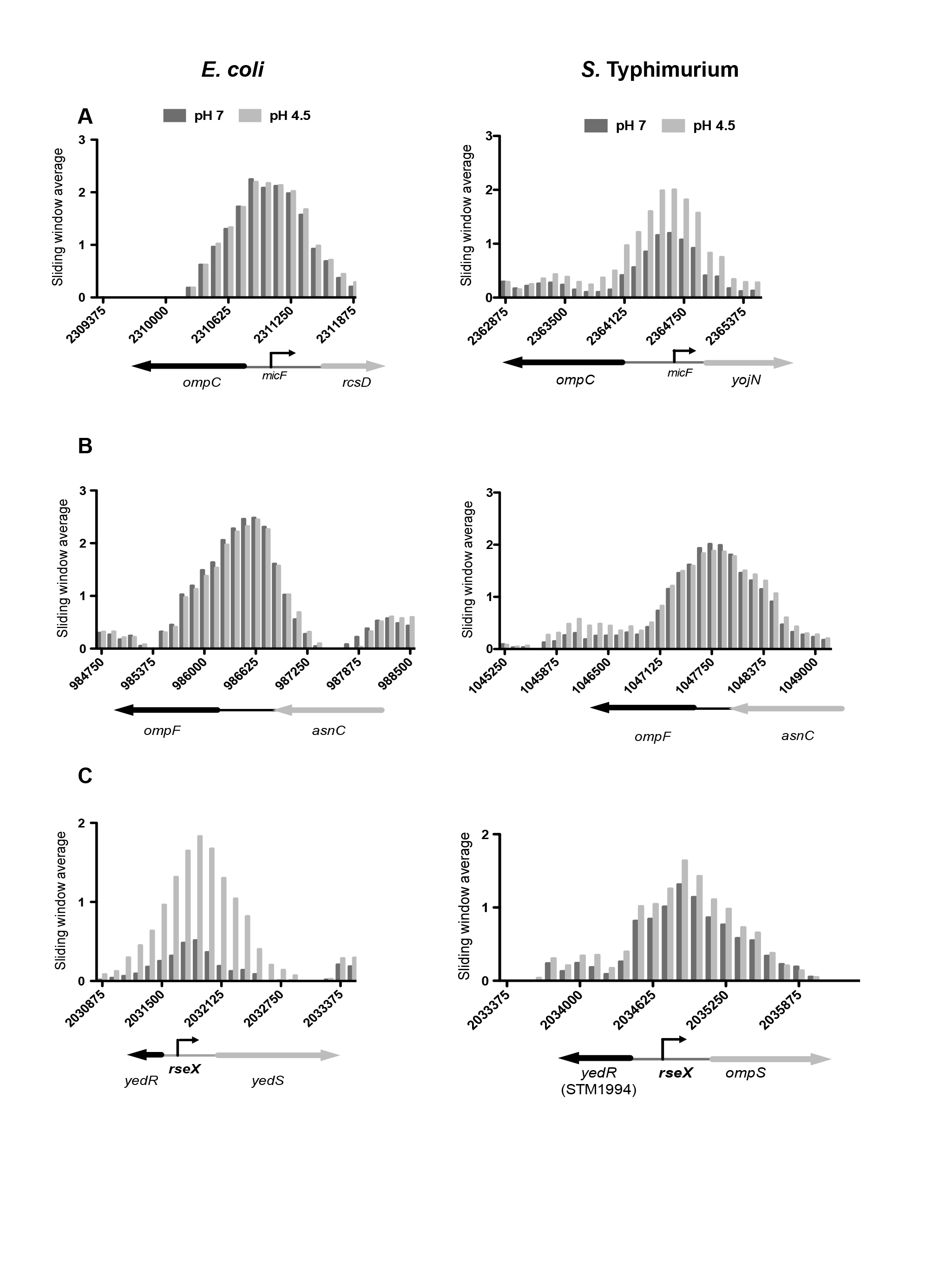

Supplement: Figure S3 — OmpR binding at genes in the core OmpR regulon. OmpR binding at pH 7 and pH 4.5 for ompC (A) ompF (B) and rseX (C) in E. coli (left panel) and S. Typhimurium (right panel). Arrows below indicate location and orientation of open reading frames. Bent arrows show small RNAs. Sliding window average of log2 enrichment as calculated by ChIPOTle [88] is shown on the y-axis. The bell-shaped curve shown here arises from DNA fragments with an OmpR binding sites located close to their centre hybridizing more frequently to the microarray than those with the binding site located toward the DNA ends. (TIF) [file pgen.1004215.s003.tif]

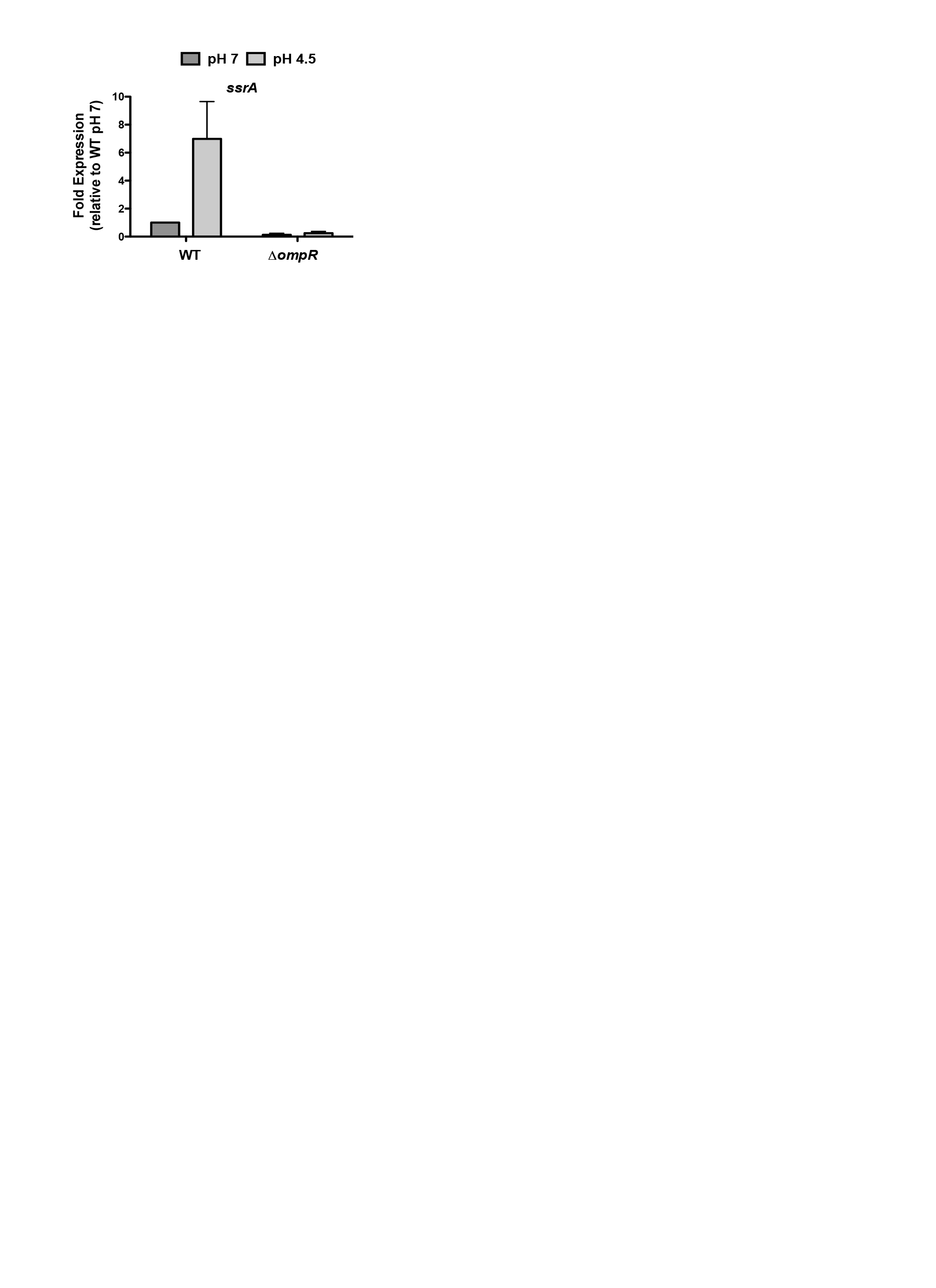

Supplement: Figure S4 — OmpR activates ssrA expression at pH 7 and pH 4.5. Quantitative PCR measurements of ssrA transcript levels at pH 7 and pH 4.5 in WT and the ΔompR mutant. N≥3; standard deviations of the mean are shown as error bars. (TIF) [file pgen.1004215.s004.tif]

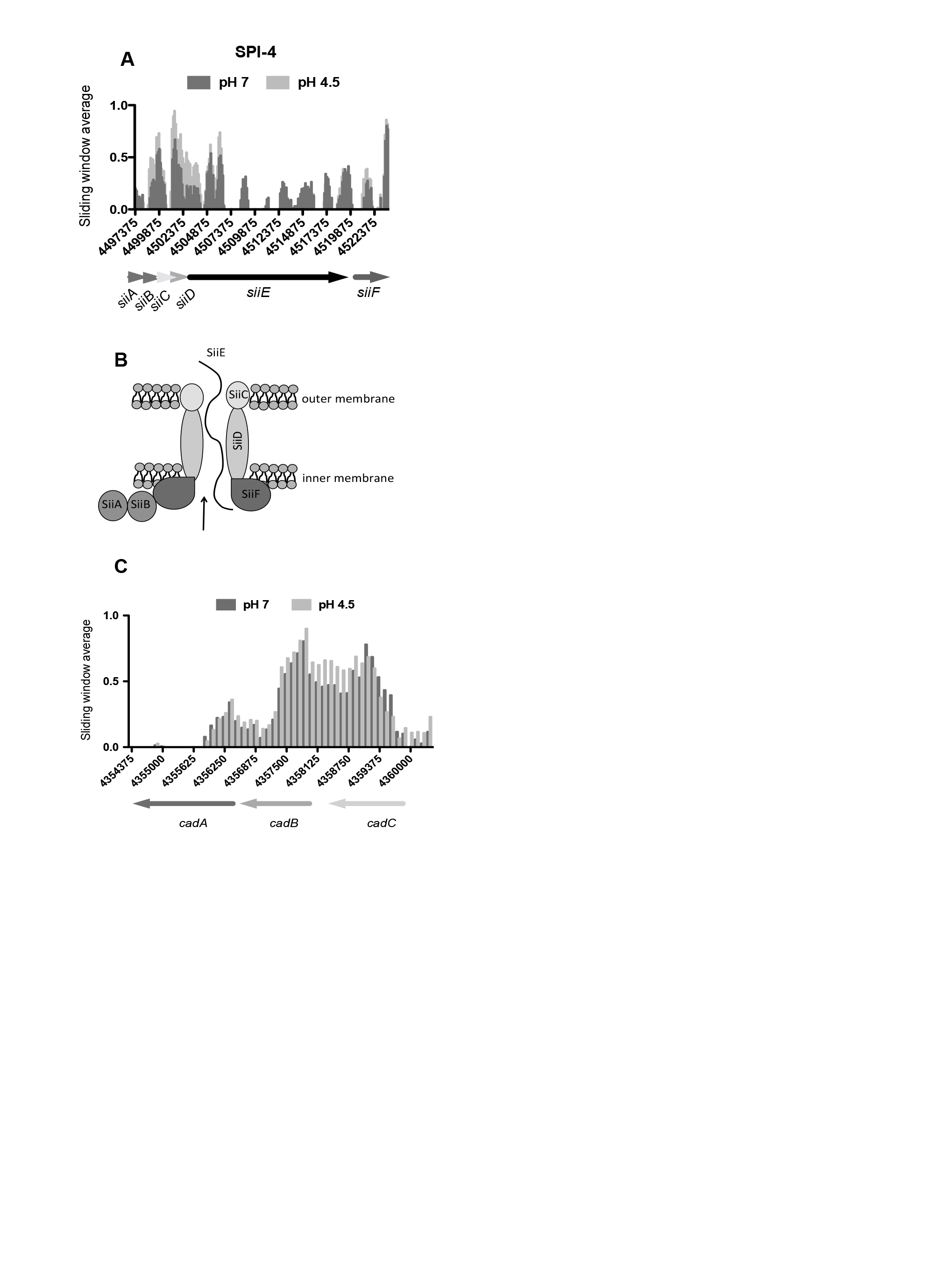

Supplement: Figure S5 — Increased OmpR binding at Salmonella pathogenicity island −4 and the cadBA operon in E. coli. Increased OmpR binding at pH 7 and pH 4.5 within SPI-4. (A) OmpR binding within SPI-4 as measured at pH 7 and pH 4.5. Arrows denote open reading frames and their orientation. Sliding window average of log2 enrichment as calculated by ChIPOTle [88] is shown on the y-axis. (B) Illustration of the SPI-4 type 1 secretion system (T1SS) and secretion of the SiiE adhesin (black curved line). SiiF is an inner membrane transporter ATPase, SiiD is a periplasmic adaptor protein and SiiC is an outer membrane protein. The functions of the SiiA and SiiC proteins are unclear; they may be interaction partners that regulate the retention of SiiE (adapted from 70). (C) Increased OmpR binding within the cadB-cadC intergenic region. Arrows denote open reading frames and their orientation. Sliding window average of log2 enrichment as calculated by ChIPOTle [88] is shown on the y-axis. (TIF) [file pgen.1004215.s005.tif]

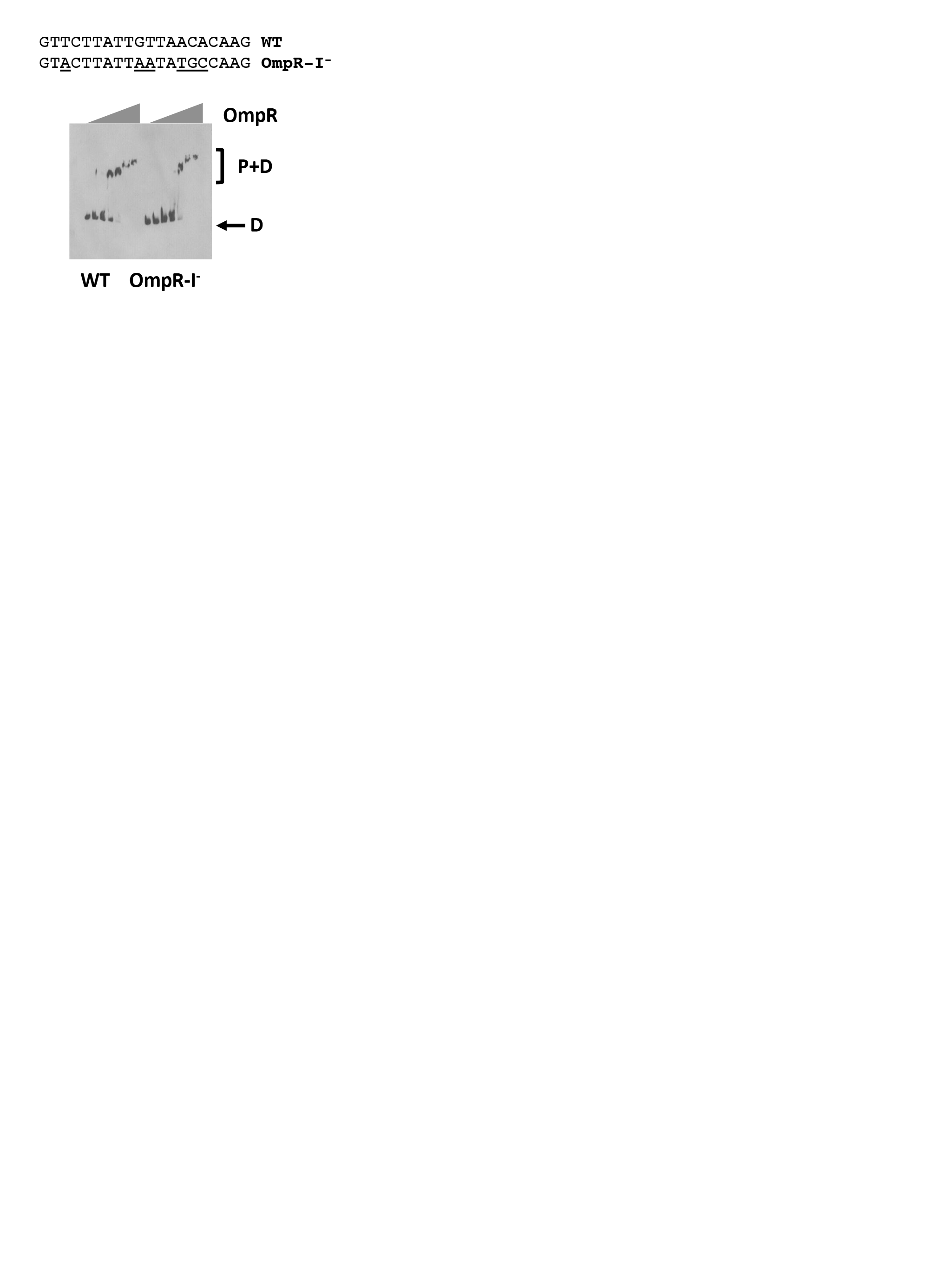

Supplement: Figure S6 — Electrophoretic mobility shift assay of OmpR binding to the phoP promoter. EMSA analysis showing OmpR binding to the wild-type phoP promoter (WT) and the phoP promoter harbouring the mutated (i.e. OmpR-I-) binding site. D, free DNA probe; P+D, protein + DNA complex. OmpR concentrations used were: 0, 0.5, 1, 2, 4, 8, and 16 µM. (TIF) [file pgen.1004215.s006.tif]
