# Supplementary material for: Bacterial Regulon Evolution: Distinct Responses and Roles for the Identical OmpR Proteins of Salmonella Typhimurium and Escherichia coli in the Acid Stress Response
Source: PLoS Genet. 2014 Mar 6;10(3):e1004215. doi: 10.1371/journal.pgen.1004215 (PMC3945435; doi:10.1371/journal.pgen.1004215)
Supplement: Table S1 — Strains and plasmids used in this study. The table provides details of the strains of Escherichia coli, Salmonella enterica serovar Typhimurium and plasmids used in the experiments described in the text. The sources of these materials or references to papers giving this information is also included. (DOCX) [file pgen.1004215.s007.docx]

**Table S1. Strains and plasmids used in this study**

| **Strain name** | **Genotype/description** | **Reference** |
| --- | --- | --- |
| *Salmonella enterica* serovar Typhimurium | | |
| SL1344 | *rpsL*, *hisG* | (3) |
| SL1344 *ompR*::3xFLAG | *ompR::*3xFLAG epitope fusion | (4) |
| SL1344 Δ*ompR* | Δ*ompR* | (4) |
| SL1344 *hns*::*kan* | *hns*::*kan*, Km^r^ | (5) |
| SL1344 *envZ*::3xFLAG | *envZ::*3xFLAG epitope fusion | This study |
| *Escherichia coli* | | |
| CSH50 | F- λ- *ara* ∆(*lac-pro*) *rpsL thi ﬁmE*::IS*1* | (6) |
| CSH50 *ompR*::3xFLAG | *ompR*::3xFLAG epitope fusion | This study |
| CSH50 Δ*ompR/envZ*::*cat* | Δ*ompR/envZ*, Cm^r^ | This study |
| CSH50 ΔP*ompR*::*cat* | ΔP*ompR*, Cm^r^ | This study |
| CSH50 P*ompR _S. enterica_* | SL1344 P*ompR* in place of native CSH50 P*ompR* | This study |
| CSH50 *ompR*/*envZ _S. enterica_* | SL1344 *ompR/envZ* in place of native CSH50 *ompR/envZ* | This study |
| CSH50 ΔP*ompRompB*::*cat* | ΔP*ompR ompR/envZ*, Cm^r^ | This study |
| CSH50 P*ompRompB _S. enterica_* | SL1344 P*ompR* and *ompB* locus in place of native CSH50 P*ompR* and *ompB* locus | This study |
| CSH50 *envZ*::3xFLAG | *envZ::*3xFLAG epitope fusion | This study |
| XL-1 | *recA1 endA1 gyrA96 thi-1 hsdR17 supE44 relA1 lac* | Stratagene |
| Plasmids | | |
| pKD46 | Ts, Amp^r^, (Carb^r^) encodes λ-Red recombinase genes | (1) |
| pKD4 | Encodes *frt-kan-frt* for gene deletions, Km^r^ | (1) |
| pKD3 | Encodes *frt-cat-frt* for gene deletions, Cm^r^ | (1) |
| pSUB11 | 3xFLAG epitope, Km^r^ | (2) |
| pCP20 | Ts, encodes FLP recombinase system, Amp^r^ (Carb^r^) | (7) |
| P*ompR S. enterica* pJET | 340 bp *ompR* promoter region from SL1344 Amp^r^ (Carb^r^), Km^r^ | This study |

Abbreviations: Ts, temperature sensitive; Cm^r^, chloramphenicol resistance; Km^r^, kanamycin resistance; Amp^r^, ampicillin resistance; Carb^r^ carbencilin resistance.

**References**

1. Datsenko KA & Wanner BL (2000) One-step inactivation of chromosomal genes in *Escherichia* *coli* K-12 using PCR products. Proc Natl Acad Sci USA 97: 6640-6645.
2. Uzzau S, Figueroa-Bossi N, Rubino S, & Bossi L (2001) Epitope tagging of chromosomal genes in *Salmonella*. Proc Natl Acad Sci USA 98: 15264-15269.
3. Hoiseth SK & Stocker BA (1981) Aromatic-dependent *Salmonella* Typhimurium are non-virulent and effective as live vaccines. Nature 291: 238-239.
4. Cameron AD & Dorman CJ (2012) A fundamental regulatory mechanism operating through OmpR and DNA topology controls expression of *Salmonella* pathogenicity islands SPI-1 and SPI-2. PLoS genetics 8: e1002615.
5. Dillon SC*,* Cameron AD, Hokamp, K, Lucchini S*,* Hinton JC, et al*.* (2010) Genome-wide analysis of the H-NS and Sfh regulatory networks in *Salmonella* Typhimurium identifies a plasmid-encoded transcription silencing mechanism. Mol Microbiol 5: 1250-1265.
6. Miller JH (1972) Experiments in Molecular Genetics. Plainview, N.Y.: Cold Spring Harbor Laboratory Press.
7. Cherepanov PP & Wackernagel W (1995) Gene disruption in *Escherichia* coli: TcR and KmR cassettes with the option of Flp-catalyzed excision of the antibiotic-resistance determinant. Gene 158: 9-14.
