# Supplementary material for: Bacterial Regulon Evolution: Distinct Responses and Roles for the Identical OmpR Proteins of Salmonella Typhimurium and Escherichia coli in the Acid Stress Response
Source: PLoS Genet. 2014 Mar 6;10(3):e1004215. doi: 10.1371/journal.pgen.1004215 (PMC3945435; doi:10.1371/journal.pgen.1004215)
Supplement: Table S2 — Oligonucleotides used in this study. The table reports the DNA sequences of primers used for cloning, quantitative PCR, mutant construction, DNase I footprinting or electrophoretic mobility shift assays (bandshifts). (DOCX) [file pgen.1004215.s008.docx]

**Table S2. Oligonucleotides used in this study**

| **Name** | **Sequence (5′-3′)** |
| --- | --- |
| *ompR_*F_pJET | CGCTCTTCACGCCAGA |
| *ompR*_R_ pJET | CGCATATCGTCATCAACC |
| *ompB*::*cat*_E.c_F | CACGCTTACAAATTGTTGCGAACCTTTGGGAGTACAAAC ATAGGAACTTCATTTAAATGG |
| *ompB*::*cat*_E.c_R | ATAGAAAGCAAAACGGGAGGCACCTTCGCCTCCCGTTTA TGCGCCTACCTGTGACGGAAG |
| *ompR*_RT_S.e_F | ATCGTCTGCTGACCCGTGAATCTT |
| *ompR*_RT_S.e_R | TTACTTTGACTACGCAGGCGACGA |
| *ompR*_E.c_RT_F | ATCGCCTGCTGACTCGTGAATCTT |
| *ompR*_E.c_RT_R | TTGCTCTGACTACGAAGACGTCGG |
| *ssrA*_RT_F | ACATAAACGGCAAAGGATGGCACG |
| *ssrA*_RT_R | TCGGGAAGTTTAACCGTCACCTCA |
| P*phoP*pJET_F | ATAGCGGCCGCAATTATATCGGTCGCGCTGT |
| P*phoP*pJET_R | GCCGTCTAGAGTGGCGTAATAATGCATTAT |
| P*phoP_*SDM_F | AACGCTAGACTGTACTTATTAATATGCCAAGGGAGAAGAG |
| P*phoP_*SDM_F | CTCTTCTCCCTTGGCATATTAATAAGTACAGTCTAGCGTT |
| RACE_*ompR* | GTTTTTGCTAGCTTAGCGACGCTTCGAACCTGGAAG |
| *phoP_*S.e_RT_F | AGTCGAGGTTCTCAGCTCCG |
| *phoP_*S.e_RT_R | CACCTGGGAGGCCAGACCGC |
| *gmk*_F_Se | AGCAAATTCGCGAAAAGATG |
| *gmk*_R_Se | TGGCAATGACTTCTTCGCTAT |
| *gmk*_F_Ec | TATTGTTTCTGCCCCCAGTG |
| *gmk*_R_Ec | GTTCGAGGAACGCATCTCTG |
| RACE_*ompR* | GTTTTTGCTAGCTTAGCGACGCTTCGAACCTGGAAG |
| JVO-0367 | ACTGACATGGAGGAGGGA |
| RNA-linker A4 | GACGAGCACGAGGACACUGACAUGGAGGAGGGAGUAGAAA |
| Bio_P*phoP*_F | GACTCTGGTCGACGAACTTAAATAA |
| Bio_P*phoP*_R | CCTCTACAACCAGTACGCGCATCAT |
| *ompR*::FLAG_E.c_F | GGGTCTGGGCTACGTCTTTGTACCGGACGGCTCTAAAGCAGACTACAAAGACCATGACGG |
| *ompR*::FLAG_E.c_R | GGGCAAATGAACTTCGTGGCGAGAAGCGCAATCGCCTCATCATATGAATATCCTCCTTAG |
| *ompB*::*cat*_E.c_F | CACGCTTACAAATTGTTGCGAACCTTTGGGAGTACAAACATAGGAACTTCATTTAAATGG |
| *ompB*::*cat*_E.c_R | ATAGAAAGCAAAACGGGAGGCACCTTCGCCTCCCGTTTATGCGCCTACCTGTGACGGAAG |
| *ompR*_conf_E.c_F | TAGGCTGAAATTCATACCAGATTT |
| *ompR*_conf_E.c_R | GGCTGGCGAACAGCAAGGTGACGATG |
| PstI_pKD4_F | GTCGATCTGCAGGTGTAGGCTGGAGCTGCTTC |
| XhoI_pKD4_R | GTCGATCTCGAGCATATGAATATCCTCCTTAG |
| P*ompR*_KO_E.c_F | GACTTGCGGCCCAGGTCACCTTTTTTGTGACCTCCGGGCGGCGCCTACCTGTGACGGAAG |
| P*ompR*_KO_E.c _R | TGTCGTCATCGACCACCAGAATCTTGTAGTTCTCTTGCATTAGGAACTTCATTTAAATGG |
| P*ompR*_int_E.c_F | GACTTGCGGCCCAGGTCACCTTTTTTGTGACCTCCGGGCGGTGTAGGCTGGAGCTGCTTC |
| P*ompR*_int_E.c_R | GCAGGCGCATGTCGTCATCGACCACCAGAATCTTGTAGTTCTCTTGCATTGTCTGTACTC |
| *ompB*-*kan*_S.e_F | GGCTCGCGTCCAGGGGACGACAAAAGAGGCATAAGAAAGGGTGTAGGCTGGAGCTGCTTC |
| *ompB*-*kan*_S.e_R | GCGTCATCCGGCGTTGAGAAGAAAGGGAGGGTAATACCTCCATATGAATATCCTCCTTAG |
| P*ompR*_KO_E.c_F | GACTTGCGGCCCAGGTCACCTTTTTTGTGACCTCCGGGCGGCGCCTACCTGTGACGGAAG |
| P*ompRompB*_KO_E.c_R | ATAGAAAGCAAAACGGGAGGCACCTTCGCCTCCCGTTTATTAGGAACTTCATTTAAATGG |
| P*ompRompB*_int_E.c_F | GACTTGCGGCCCAGGTCACCTTTTTTGTGACCTCCGGGCGCTCTTCACGCCAGAGATAAT |
| *ompR* _F | CACGCTTACAAATTGTTGCGAACCTTTGGGAGTACAAACAATGCAAGAGAATTATAAGAT |
| *kan*_R | TATCTTATAGAAAGCAAAACGGGAGGCACCTTCGCCTCCCCATATGAATATCCTCCTTAG |
| Bio_P*phoP*_F | GACTCTGGTCGACGAACTTAAATAA |
| Bio_P*phoP*_R | CCTCTACAACCAGTACGCGCATCAT |
| P*mgtC*_F_EM | GAACCCATTTTTTCCTCGTCATGTT |
| P*mgtC*_R_EM | TACGTTCCTCCATTTTTTCTGGAAG |
| *mgtC*_RT_F | CGCAGATTGTCTCTGGGATT |
| *mgtC*_RT_R | GGCGCAAAGAATAATGATCG |
| PompC_EM_F | ATAAATCAGCCGGGTGTGTC |
| PompC_EM_R | GACAGTACTTTAACTTTCAT |
| *Kan_*bio_F | CAAGATGGATTGCACG |
| *Kan_*bio_R | TCATCCTGATCGACAAGACC |
| *envZ*::FLAG_E.c_F | AGTGCCGGTAACGCGGGCGCAGGGCACGACAAAAGAAGGGGACTACAAAGACCATGACGG |
| *envZ*::FLAG_E.c_R | ATAGAAAGCAAAACGGGAGGCACCTTCGCCTCCCGTTTATCATATGAATATCCTCCTTAG |
| *envZ*::FLAG_S.T_F | GGTTCCTGTGGCTCGCGTCCAGGGGACGACAAAAGAGGCAGACTACAAAGACCATGACGG |
| envZ::FLAG_S.T_R | TCCGGCGTTGAGAAGAAAGGGAGGGTAATACCTCCCTTTCCATATGAATATCCTCCTTAG |
| *envZ*_RT_F_S.T | CCTACCTGGTGGTGCTGAAC |
| *envZ*_RT_R_S.T | CCAGTTGCAGCTTATCGGTC |
| *envZ*_RT_F_E.c | CCAGCCTGGTGACGACTTAT |
| *envZ*_RT_R_E.c | ATCAACATACGCACTTCGTA |
