# Supplementary material for: Bacterial Regulon Evolution: Distinct Responses and Roles for the Identical OmpR Proteins of Salmonella Typhimurium and Escherichia coli in the Acid Stress Response
Source: PLoS Genet. 2014 Mar 6;10(3):e1004215. doi: 10.1371/journal.pgen.1004215 (PMC3945435; doi:10.1371/journal.pgen.1004215)
Supplement: Table S3 — Common OmpR targets in SL1344 and CSH50. The table lists those genes that are common to S. Typhimurium and E. coli and that were bound by OmpR protein in ChIP-chip experiments. (DOCX) [file pgen.1004215.s009.docx]

**Table S3. Common OmpR targets in SL1344 and CSH50**

| **No.** | **SL1344 gene**  **name** | **CSH50**  **gene**  **name** | **Function** | **SL1344 Peak**  **start** | **CSH50**  **Peak**  **start** |
| --- | --- | --- | --- | --- | --- |
| **1** | *bcfA* | *elfA* (*ycb)* | predicted fimbrial-like adhesin protein | 25000 | 997125 |
| **2** | STM0291 | *rhsA* | *rhsA* element core protein RshA | 334625 | 3764125 |
| **3** | *ybiF* | *rhtA* | threonine and homoserine efflux system | 898250 | 849375 |
| **4** | *ompF* | *ompF* | outer membrane porin 1a | 1047125 | 985750 |
| **5** | *csgD* | *csgD* | DNA-binding transcriptional activator for *csgBA* | 1186125 | 1102250 |
| **6** | *flhD* | *flhD* | DNA-binding transcriptional dual regulator with FlhC | 1979625 | 1976000 |
| **7** | *ompC* | *ompC* | outer membrane porin protein C | 2364125 | 2310500 |
| **8** | *lrhA, ala* | *lrhA, alaA* | DNA-binding transcriptional repressor of flagellar, motility and chemotaxis genes, | 2438750 | 2405125 |
| **9** | *ygjU* | *sstT* | sodium:serine/threonine symporter | 3415250 | 3237875 |
| **10** | *yqjA* | *exuR* | DNA-binding transcriptional repressor | 3415625 | 3245500 |
| **11** | *ompR* | *ompR* | DNA-binding response regulator in two-component regulatory system with EnvZ | 3681875 | 3534375 |
| **12** | *ydeV* | *lsrK* | autoinducer-2 (AI-2) kinase | 4302125 | 1597000 |
| **13** | STM1994 | *rseX* | ncRNA | 2034500 | 2031625 |
| **14** | STM2503 | *yfgF* | cyclic-di-GMP phosphodiesterase, anaerobic | 2618625 | 2626500 |
| **15** | *mlc* | *dgsA* | Global DNA-binding transcriptional repressor; for anaerobic growth on glucosamine | 1523125 | 1665750 |
