# Supplementary material for: Bacterial Regulon Evolution: Distinct Responses and Roles for the Identical OmpR Proteins of Salmonella Typhimurium and Escherichia coli in the Acid Stress Response
Source: PLoS Genet. 2014 Mar 6;10(3):e1004215. doi: 10.1371/journal.pgen.1004215 (PMC3945435; doi:10.1371/journal.pgen.1004215)
Supplement: Table S4 — PhoP-regulated genes identified as OmpR targets. The table lists the genes that are known to be regulated by the PhoP protein in S. Typhimurium and that were bound by the OmpR protein in the ChIP-chip experiments. (DOCX) [file pgen.1004215.s010.docx]

**Table S4. PhoP-regulated genes identified as OmpR targets**

| **No.** | **Gene name** | **Function** | **Peak start** |
| --- | --- | --- | --- |
| **1** | intergenic *mgtC* | Mg^2+^ transport protein | 3986625 |
| **2** | intergenic *mig*-*14* | transcriptional activator | 2952000 |
| **3** | intergenic *prgH* | needle complex inner membrane protein | 3040125 |
| **4** | intergenic *pmrD* | polymyxin resistance protein B | 2409000 |
| **5** | intergenic *pgtC* | phosphoglycerate transport regulatory protein precursor | 2509375 |
| **6** | intergenic *slyB* | Outer membrane lipoprotein | 1477625 |
| **7** | intergenic *yaiB* (*iraP*) | Hypothetical protein | 436375 |
| **8** | *pagO* | integral membrane protein | 1917500 |
| **9** | *pagK* | PagK | 1920750 |
| **10** | *prgJ* | needle complex minor subunit | 3038375 |
| **11** | *prgI* | needle complex major subunit | 3038750 |
| **12** | *pqaA* | PhoPQ-regulated protein | 1577875 |
| **13** | *ssrB* | transcriptional activator | 1432875 |
