# Supplementary material for: Bacterial Regulon Evolution: Distinct Responses and Roles for the Identical OmpR Proteins of Salmonella Typhimurium and Escherichia coli in the Acid Stress Response
Source: PLoS Genet. 2014 Mar 6;10(3):e1004215. doi: 10.1371/journal.pgen.1004215 (PMC3945435; doi:10.1371/journal.pgen.1004215)
Supplement: Table S5 — List of OmpR sites used to build OmpR weight matrix. The table lists the DNA sequences of those OmpR binding sites that were used to construct the weight matrix for OmpR. (DOCX) [file pgen.1004215.s011.docx]

**Table S5. List of OmpR sites used to build OmpR weight matrix**

| **Promoter** | **OmpR binding site sequence** |
| --- | --- |
| [*bolA*](http://regulondb.ccg.unam.mx/operon?organism=ECK12&term=ECK120014663&format=jsp&type=operon) | CTAAATATTTGTTGTTAAGC |
| [*csgD*](http://regulondb.ccg.unam.mx/operon?organism=ECK12&term=ECK120014668&format=jsp&type=operon) | GTTACATTTAGTTACATGTT |
| [*cnB*](http://regulondb.ccg.unam.mx/operon?organism=ECK12&term=ECK120028089&format=jsp&type=operon) | GCAAACATAAATAACATTAC |
| [*fadL*](http://regulondb.ccg.unam.mx/operon?organism=ECK12&term=ECK120014891&format=jsp&type=operon) | GTAACATAGTTTGTATAAAA |
| [*fadL*](http://regulondb.ccg.unam.mx/operon?organism=ECK12&term=ECK120014891&format=jsp&type=operon) | GAAAACCCTGTTTACAAAGT |
| [*flhD*](http://regulondb.ccg.unam.mx/operon?organism=ECK12&term=ECK120014934&format=jsp&type=operon) | GGGCATTATCTGAACATAAA |
| [*micF*](http://regulondb.ccg.unam.mx/operon?organism=ECK12&term=ECK120014444&format=jsp&type=operon) | AAAAGTTTTAGTATCATATT |
| [*nmpC*](http://regulondb.ccg.unam.mx/operon?organism=ECK12&term=ECK120014583&format=jsp&type=operon) | GAAACCAAAACTTACATCTT |
| [*ompC*](http://regulondb.ccg.unam.mx/operon?organism=ECK12&term=ECK120014518&format=jsp&type=operon) | TTTACATTTTGAAACATCTA |
| [*ompC*](http://regulondb.ccg.unam.mx/operon?organism=ECK12&term=ECK120014518&format=jsp&type=operon) | AGCGATAAATGAAACATCTT |
| [*ompC*](http://regulondb.ccg.unam.mx/operon?organism=ECK12&term=ECK120014518&format=jsp&type=operon) | AAAAGTTTTAGTATCATATT |
| [*ompF*](http://regulondb.ccg.unam.mx/operon?organism=ECK12&term=ECK120014863&format=jsp&type=operon) | GTTACGGAATATTACATTGC |
| [*ompF*](http://regulondb.ccg.unam.mx/operon?organism=ECK12&term=ECK120014863&format=jsp&type=operon) | TTTACTTTTGGTTACATATT |
| [*ompF*](http://regulondb.ccg.unam.mx/operon?organism=ECK12&term=ECK120014863&format=jsp&type=operon) | TTTTCTTTTTGAAACCAAAT |
| [*ompF*](http://regulondb.ccg.unam.mx/operon?organism=ECK12&term=ECK120014863&format=jsp&type=operon) | CTTTATCTTTGTAGCACTTT |
| [*omrA*](http://regulondb.ccg.unam.mx/operon?organism=ECK12&term=ECK120023636&format=jsp&type=operon) | TACACACCTCGTTGCATTTC |
| [*omrB*](http://regulondb.ccg.unam.mx/operon?organism=ECK12&term=ECK120023635&format=jsp&type=operon) | CAAACCTTTGGTTACACTTT |
| *tppB* | GTAACAGATTATTACAAAGG |
| *hilC* | GTATCTTTTTGTGACGAAAT |
