# Supplementary material for: Bacterial Regulon Evolution: Distinct Responses and Roles for the Identical OmpR Proteins of Salmonella Typhimurium and Escherichia coli in the Acid Stress Response
Source: PLoS Genet. 2014 Mar 6;10(3):e1004215. doi: 10.1371/journal.pgen.1004215 (PMC3945435; doi:10.1371/journal.pgen.1004215)
Supplement: Table S6 — List of OmpR sites found in the ChIP-on-chip data sets for SL1344 and CSH50 at pH 7 and pH 4.5. The table lists the genes in S. Typhimurium and E. coli that were bound by the OmpR protein in the ChIP-chip experiments. (DOCX) [file pgen.1004215.s012.docx]

**Table S. 6. List of OmpR sites found in the ChIP-on-chip data sets for SL1344 and CSH50 at pH 7 and pH 4.5**

| **SL1344 pH 7** | | | | | | |
| --- | --- | --- | --- | --- | --- | --- |
| **No.** | **Seq_id** | **Strand** | **Start** | **End** | **Sequence** | **Weight** |
| 1 | *adrA* | R | -399 | -380 | GGAACATTTAGAAACAATTT | 10.0 |
| 2 | STM0557 | R | -399 | -380 | GCAAAAATATGTAACAACGT | 7.7 |
| 3 | STM0557 | D | -404 | -385 | TATAAACGTTGTTACATATT | 7.2 |
| 4 | *cstA* | D | -536 | -517 | GAAACAAAATGTAACATCTC | 9.9 |
| 5 | *cstA* | R | -541 | -522 | GTTACATTTTGTTTCTATAA | 7.6 |
| 6 | *tatE* | R | -259 | -240 | GTAACTTCTTGGTACCAAAC | 7.5 |
| 7 | *ompF* | R | -993 | -974 | ATAACATTTGTTTACATATT | 10.9 |
| 8 | *ompF* | D | -667 | -648 | ATTAATTTTATTTACTTATT | 8.4 |
| 9 | *yccT* | R | -158 | -139 | GAAAAATTTCGATACATTAT | 9.3 |
| 10 | *csgD* | R | -919 | -900 | TTTACATTTGGTTACAAGTT | 9.8 |
| 11 | *csgD* | D | -914 | -895 | GTAACCAAATGTAAAAATAT | 7.2 |
| 12 | *tppB* | R | -562 | -543 | GTAACAGATTATTACAAAGC | 9.4 |
| 13 | *sopE2* | D | -400 | -381 | TTAACTGATTTTTATATATT | 7.8 |
| 14 | STM1994 | D | -358 | -339 | AAATACCTTTGTTACATGTT | 7.8 |
| 15 | STM1994 | D | -378 | -359 | GAAACAAATTGAAATATTTT | 7.5 |
| 16 | *ompC* | R | -588 | -569 | AAAAGTTTTAGTATCATATT | 9.7 |
| 17 | *fadL* | D | -191 | -172 | GTTACAGCATGTAACATAGT | 9.7 |
| 18 | *fadL* | D | -132 | -113 | GAAAACCCTGTTTACAAAGT | 7.6 |
| 19 | *orgC* | D | -203 | -184 | TCAACTTTTGCTAACATGTT | 7.2 |
| 20 | *ygjT* | D | -126 | -107 | CAAACACTTTGTTACATCCT | 7.8 |
| 21 | *yqjA* | D | -410 | -391 | GTTACAATTAGATGCATTTT | 9.4 |
| 22 | *yqjA* | D | -420 | -401 | GTTGACCTTTGTTACAATTA | 7.8 |
| 23 | *yqjA* | R | -415 | -396 | GCATCTAATTGTAACAAAGG | 7.2 |
| 24 | *yhjD* | D | -454 | -435 | TTTAGCTTTTGTTACACATT | 10.1 |
| **SL1344 pH 4.5** | | | | | | |
| **No.** | **Seq_id** | **Strand** | **Start** | **End** | **Sequence** | **Weight** |
| 1 | *adrA* | R | -524 | -505 | GGAACATTTAGAAACAATTT | 10.0 |
| 2 | STM0557 | R | -524 | -505 | GCAAAAATATGTAACAACGT | 7.7 |
| 3 | STM0557 | D | -529 | -510 | TATAAACGTTGTTACATATT | 7.2 |
| 4 | *cstA* | D | -536 | -517 | GAAACAAAATGTAACATCTC | 9.9 |
| 5 | *cstA* | R | -541 | -522 | GTTACATTTTGTTTCTATAA | 7.6 |
| 6 | *tatE* | R | -759 | -740 | GTAACTTCTTGGTACCAAAC | 7.5 |
| 7 | STM0721 | R | -486 | -467 | GAGACACATTGTGACAAATA | 7.9 |
| 8 | STM0854 | D | -576 | -557 | GTAACAATAAATTACATGTT | 8.6 |
| 9 | STM0854 | R | -581 | -562 | GTAATTTATTGTTACAATAA | 7.5 |
| 10 | STM0856 | R | -205 | -186 | GTTACTTTTATTAACATGTT | 10.5 |
| 11 | STM0858 | D | -468 | -449 | TGCAATTTTATTTACATAAT | 7.9 |
| 12 | *ompF* | R | -993 | -974 | ATAACATTTGTTTACATATT | 10.9 |
| 13 | *ompF* | D | -667 | -648 | ATTAATTTTATTTACTTATT | 8.4 |
| 14 | STM2626 | D | -408 | -389 | CTTGCCTATCGTAACATCAT | 7.6 |
| 15 | *ompA* | D | -60 | -41 | TTTACTTAATGATACAAATT | 9.7 |
| 16 | *yccT* | R | -658 | -639 | GAAAAATTTCGATACATTAT | 9.3 |
| 17 | STM1109 | D | -693 | -674 | GAAAATCTGAGAAACATAAC | 7.3 |
| 18 | *wrbA* | R | -634 | -615 | ATTAATTATTGTTATAAATC | 7.7 |
| 19 | *csgF* | R | -1169 | -1150 | TTTACATTTGGTTACAAGTT | 9.8 |
| 20 | *csgF* | D | -1164 | -1145 | GTAACCAAATGTAAAAATAT | 7.2 |
| 21 | *envF* | R | -70 | -51 | TAAAATACATGTTACATATA | 8.2 |
| 22 | *cspH* | R | -222 | -203 | GTGACCTCTTTTATCATATA | 7.2 |
| 23 | *tppB* | R | -687 | -668 | GTAACAGATTATTACAAAGC | 9.4 |
| 24 | *yddG* | R | -606 | -587 | GTTTCATAAGATTACAAATT | 7.9 |
| 25 | STM1575 | D | -872 | -853 | TTTACAAATAGTAACAATTA | 8.5 |
| 26 | STM1650 | D | -324 | -305 | TTAACATTTATTGGCAATTT | 7.9 |
| 27 | STM1839 | D | -1009 | -990 | ATAAAACTATGTTGCATATA | 8.0 |
| 28 | STM1839 | R | -1220 | -1201 | TTTTATCATTGTGACAATTT | 7.4 |
| 29 | *sopE2* | D | -650 | -631 | TTAACTGATTTTTATATATT | 7.8 |
| 30 | STM1861 | D | -41 | -22 | AATTCATAATGTTACATATT | 9.1 |
| 31 | *sirA* | R | -278 | -259 | GTTAATTATTGTTACAAAGT | 12.0 |
| 32 | *yodD* | D | -413 | -394 | GTAAATATTTTTTACATGAA | 8.1 |
| 33 | STM1994 | D | -608 | -589 | AAATACCTTTGTTACATGTT | 7.8 |
| 34 | STM1994 | D | -628 | -609 | GAAACAAATTGAAATATTTT | 7.5 |
| 35 | *cspB* | R | -59 | -40 | TAATATTTTTTTAACAAAAC | 7.6 |
| 36 | *rfbM* | D | -278 | -259 | GACAATTTTTTTAACATCTT | 10.0 |
| 37 | *sseK2* | D | -167 | -148 | CAAAATTATAGAAACATAAA | 8.2 |
| 38 | *setB* | D | -980 | -961 | GTTTCCGTTTGTAACATATT | 11.3 |
| 39 | *ompC* | R | -838 | -819 | AAAAGTTTTAGTATCATATT | 9.7 |
| 40 | *lrhA* | D | -101 | -82 | GTAAATGCTTTTTAAATATT | 7.2 |
| 41 | *yfcZ* | D | -566 | -547 | GTTACAGCATGTAACATAGT | 9.7 |
| 42 | *yfcZ* | D | -507 | -488 | GAAAACCCTGTTTACAAAGT | 7.6 |
| 43 | STM2406 | R | -281 | -262 | GTAAAGATTTGTTTCATCGC | 8.1 |
| 44 | *perM* | R | -232 | -213 | GTGACATTTCGATACAATTC | 8.2 |
| 45 | *gogB* | D | -179 | -160 | ATTACCTTTAGTAACAAACC | 7.4 |
| 46 | *gogB* | D | -371 | -352 | AAAAAATTAAGTATCAACTT | 7.3 |
| 47 | *pipB2* | D | -737 | -718 | TTTACGTCTGGTTACATAAT | 9.1 |
| 48 | *pipB2* | D | -190 | -171 | ATAAATTTTTATTACGTTGC | 7.2 |
| 49 | *mig-14* | D | -163 | -144 | CTAAACATTTGTCACATTTT | 7.7 |
| 50 | *orgC* | R | -924 | -905 | GTATCTTTTTGTGACGAAAT | 9.1 |
| 51 | *orgC* | R | -884 | -865 | GTAAGCTTTCATTACAAAAT | 9.1 |
| 52 | *orgC* | D | -578 | -559 | TCAACTTTTGCTAACATGTT | 7.2 |
| 53 | *cysC* | R | -162 | -143 | TGAACAATTAGTAACAAATT | 10.2 |
| 54 | *stdA* | D | -1662 | -1643 | GAAATATTTTGTAATATATT | 7.2 |
| 55 | STM3052 | D | -569 | -550 | GAAACTATTTGTAACCACAT | 9.4 |
| 56 | STM3052 | D | -615 | -596 | GGAAATTTTTGTAACTATAT | 9.1 |
| 57 | *ygjT* | D | -251 | -232 | CAAACACTTTGTTACATCCT | 7.8 |
| 58 | *ygjU* | D | -410 | -391 | GTTACAATTAGATGCATTTT | 9.4 |
| 59 | *ygjU* | D | -420 | -401 | GTTGACCTTTGTTACAATTA | 7.8 |
| 60 | *ygjU* | R | -415 | -396 | GCATCTAATTGTAACAAAGG | 7.2 |
| 61 | *tdcA* | D | -227 | -208 | GAAACATTTAATAAAATTTT | 8.3 |
| 62 | *tdcA* | D | -258 | -239 | GTCACATTTTTTTACCTTAT | 8.2 |
| 63 | *yhjD* | D | -454 | -435 | TTTAGCTTTTGTTACACATT | 10.1 |
| 64 | *yhjK* | R | -507 | -488 | CTCAAACTTTTTAACATTTT | 8.2 |
| 65 | *dppA* | R | -150 | -131 | TTACAATTTTGTGACATATT | 9.4 |
| 66 | *rhuM* | R | -176 | -157 | AGAAAATTTTGTAACTTAAT | 8.5 |
| 67 | *idnD* | R | -250 | -231 | GTCACAAATAGTGACAAATA | 8.5 |
| 68 | *idnD* | D | -245 | -226 | GTCACTATTTGTGACTTATG | 8.1 |
| **CSH50 pH 7** | | | | | | |
| **No.** | **Seq_id** | **Strand** | **Start** | **End** | **Sequence** | **Weight** |
| 1 | *yafT* | R | -307 | -288 | CTTTGTTTTTGTTACAAGTC | 7.2 |
| 2 | *gltA* | D | -482 | -463 | GGTAATGTTTGTAACAACTT | 9.6 |
| 3 | *ompF* | R | -730 | -711 | TTTACTTTTGGTTACATATT | 12.6 |
| 4 | *ompF* | R | -740 | -721 | GTTACATATTTTTTCTTTTT | 7.6 |
| 5 | *ompF* | D | -1067 | -1048 | GTTACCCTTGGAAAAATAAT | 7.3 |
| 6 | *csgD* | R | -644 | -625 | GTTACATTTAGTTACATGTT | 11.5 |
| 7 | *csgD* | D | -639 | -620 | GTAACTAAATGTAACTCGTT | 7.2 |
| 8 | *yehA* | R | -72 | -53 | TAAAATTAAATTTACATTTT | 7.7 |
| 9 | *ompC* | R | -736 | -717 | AAAAGTTTTAGTATCATATT | 9.4 |
| 10 | *ompC* | R | -695 | -676 | TTTACATTTTGAAACATCTA | 9.1 |
| 11 | *sstT* | D | -240 | -221 | CAAACACTTTGTTACATCCT | 7.6 |
| 12 | *exuR* | R | -269 | -250 | GAATCTAATTGTAACAAAGG | 8.5 |
| 13 | *exuR* | D | -264 | -245 | GTTACAATTAGATTCAATTT | 8.3 |
| 14 | *exuR* | D | -274 | -255 | GTTGACCTTTGTTACAATTA | 7.6 |
| 15 | *yhjB* | R | -316 | -297 | TTAAGTCTTTTTAACGTATT | 7.4 |
| 16 | *yiaG* | D | -136 | -117 | AAAAATAATTGTTGCATCAC | 7.5 |
| 17 | *yibB* | D | -190 | -171 | GATAGCTTTATTTACAAAAT | 8.4 |
| 18 | *aspA* | D | -181 | -162 | GTTACCTTTTATTGCCATTT | 7.8 |
| **CSH50 pH 4.5** | | | | | | |
| **No.** | **Seq_id** | **Strand** | **Start** | **End** | **Sequence** | **Weight** |
| 1 | *gltA* | D | -357 | -338 | GGTAATGTTTGTAACAACTT | 9.6 |
| 2 | *ompF* | R | -730 | -711 | TTTACTTTTGGTTACATATT | 12.6 |
| 3 | *ompF* | R | -740 | -721 | GTTACATATTTTTTCTTTTT | 7.6 |
| 4 | *ompF* | D | -1067 | -1048 | GTTACCCTTGGAAAAATAAT | 7.3 |
| 5 | *csgD* | R | -769 | -750 | GTTACATTTAGTTACATGTT | 11.5 |
| 6 | *csgD* | D | -764 | -745 | GTAACTAAATGTAACTCGTT | 7.2 |
| 7 | *yehA* | R | -72 | -53 | TAAAATTAAATTTACATTTT | 7.7 |
| 8 | *ompC* | R | -611 | -592 | AAAAGTTTTAGTATCATATT | 9.4 |
| 9 | *ompC* | R | -570 | -551 | TTTACATTTTGAAACATCTA | 9.1 |
| 10 | *exuR* | R | -269 | -250 | GAATCTAATTGTAACAAAGG | 8.5 |
| 11 | *exuR* | D | -264 | -245 | GTTACAATTAGATTCAATTT | 8.3 |
| 12 | *exuR* | D | -274 | -255 | GTTGACCTTTGTTACAATTA | 7.6 |
| 13 | *yiaG* | D | -136 | -117 | AAAAATAATTGTTGCATCAC | 7.5 |
| 14 | *yibB* | D | -190 | -171 | GATAGCTTTATTTACAAAAT | 8.4 |
| 15 | *aspA* | D | -181 | -162 | GTTACCTTTTATTGCCATTT | 7.8 |
