# Supplementary material for: Bacterial Regulon Evolution: Distinct Responses and Roles for the Identical OmpR Proteins of Salmonella Typhimurium and Escherichia coli in the Acid Stress Response
Source: PLoS Genet. 2014 Mar 6;10(3):e1004215. doi: 10.1371/journal.pgen.1004215 (PMC3945435; doi:10.1371/journal.pgen.1004215)
Supplement: Text S1 — This text provides details of the construction of mutant bacterial strains in which components of the ompB locus from S. Typhimurium were transferred to E. coli, the production of genes expressing FLAG-tagged OmpR and EnvZ proteins and a description of the site-directed mutagenesis of the regulatory region of the phoP gene in S. Typhimurium. Relevant references are also included. (DOCX) [file pgen.1004215.s013.docx]

**Cloning and mutant construction**

CSH50 *ompR*::x3FLAG

To map OmpR distribution in *E. coli* a 3xFLAG epitope tag was fused to the C-terminus of OmpR in strain CSH50. CSH50 *ompR*::3xFLAG was generated in an identical manner as SL1344 *ompR*::3xFLAG using a modified version of the λ Red recombination system (1, 2). The primers *ompR*::FLAG_E.c_F and *ompR*::FLAG_E.c_R (Table S2) were used to amplify the FLAG epitope and kanamycin resistance marker from the plasmid pSUB11. The *ompR/envZ* locus (*ompB*) has a four base pair overlap between the *ompR* stop codon and *envZ* start codon therefore the reverse primer (*ompR*::FLAG_E.c_R; Table S2) was designed to reinstate the natural *envZ* start codon to avoid disrupting translation initiation in *envZ*. Insertion of this DNA fragment by λ Red recombination removed the *ompR* stop codon and inserted the FLAG epitope fusion in-frame with the *ompR* gene. The *ompR*::3xFLAG mutation was transduced into a fresh background (CSH50) by P1 transduction and the kanamycin resistance cassette was removed using the plasmid pCP20. Correct structure of the epitope fusion was confirmed by PCR using primers *ompR_*conf_E.c_F and *ompR*_conf_E.c_R (Table S2) followed by DNA sequencing and western blot analysis. The kanamycin cassette was subsequently removed using the plasmid pCP20.

CSH50 *envZ*::x3FLAG and SL1344 *envZ*::x3FLAG

The strain CSH50 *envZ*::x3FLAG was generated using a modified version of the λ Red recombination method (1, 2). The primers *envZ*::FLAG_E.c_F and *envZ*::FLAG_E.c_R (Table S2) were used to amplify the FLAG epitope and kanamycin resistance marker from the plasmid pSUB11. The *envZ*::3xFLAG mutation was transduced into a fresh background (CSH50) by P1 transduction. The strain SL1344 *envZ*::x3FLAG was created in the same manner as CSH50 *envZ*::x3FLAG except using the primers *envZ*::FLAG_S.T_F and *envZ*::FLAG_S.T_R (Table S2). This mutation was marker rescued into SL1344 by P22 transduction.

CSH50 P*ompR_S. enterica_*

As the *ompR* regulatory region of both SL1344 and CSH50 share 88% DNA sequence identity, it was necessary to first delete the *ompR* regulatory region in CSH50 to avoid unwanted homologous recombination occurring between the *ompR* regulatory regions from both strains. The *cat* gene was amplified from plasmid pKD3 using primers P*ompR*_KO_E.c_F and P*ompR*_KO_E.c_R (Table S2) and integrated into CSH50 using the λ Red recombination system (1) creating CSH50 ΔP*ompR*::*cat*. The *ompR* regulatory region of SL1344 was amplified from plasmid Kan^R^ P*ompR _S. enterica_* pJET using primers P*ompR_*int_E.c_F and P*ompR_*int_E.c_R (see Table S2) and integrated into CSH50 ΔP*ompR*::*cat*. This replaced the *cat* gene with the *ompR* regulatory region from SL1344 and upstream *kan* cassette. Transformants were screened by checking resistance to kanamycin and sensitivity to chloramphenicol on L-agar plates containing either chloramphenicol or kanamycin. Subsequently, correct integration was confirmed by PCR and DNA sequencing. The mutant was P1 transduced into a fresh background (CSH50) and the kanamycin cassette was then removed using the plasmid pCP20 and screened for sensitivity to kanamycin.

CSH50 *ompB _S. enterica_*

To create CSH50 *ompB _S. enterica_* the *ompB* locus of CSH50 was replaced by the *cat* using primers *ompB*::*cat*_E.c_F and *ompB*::*cat_*E.c_R and the λ Red recombination system creating CSH50 Δ*ompB*::*cat*. The *kan* gene was then introduced downstream of the *ompB* locus in SL1344 using the primers *ompB*_*kan*_S.e_F and *ompB*_*kan*_S.e_R (Table S2) creating SL1344 *ompB-kan.* Next the *ompB-kan* locus was amplified using primers *ompR*_F and *kan*_R (Table S2) and this product was then integrated into CSH50 Δ*ompB*::*cat* by λ Red recombination. This introduced the SL1344 *ompB-kan* locus downstream of the native *ompR* promoter in CSH50. Transformants were screened for resistance to kanamycin and sensitivity to chloramphenicol. Integration was confirmed by PCR and DNA sequencing. The mutant was then moved into a fresh background by P1 transduction and the *kan* gene was removed using the plasmid pCP20.

CSH50 P*ompRompB_s.enterica_*

The *ompR* promoter and *ompB* locus in CSH50 were replaced with a chloramphenicol resistance cassette using the primers P*ompRompB*_K.O_E.c_F and P*ompRompB*_K.O_E.c_R (Table S2). In SL1344 a kanamycin resistance cassette was introduced downstream of the *ompB* locus using the primers *ompB_kan*_S.e_F and *ompB_kan*_S.e_R (Table S2). The *ompR* regulatory region, *ompB* locus and the kanamycin cassette were amplified using the primers P*ompRompB*_int_F and *kan*_R (Table S2). This PCR product was introduced into strain CSH50 P*ompRompB*::*cat* and integrated in place of the *cat* gene. Transformants were screened by checking resistance to kanamycin and sensitivity to chloramphenicol. Positive transformants were verified by PCR and DNA sequencing. The mutant was P1 transduced into a fresh background and the kanamycin cassette was removed using the plasmid pCP20.

**Site-directed mutagenesis**

Site-directed mutagenesis (SDM) of the OmpR-I binding site in the *phoP* promoter was carried out using the QuikChange II SDM kit (Stratagene) according to the manufacturer’s instructions. The plasmid P*phoP*pJET was used as template and the primers P*phoP*_SDM_F and P*phoP*_SDM_R Table S2 which introduced 6 bp substitutions into the OmpR-I site. Plasmid DNA was subsequently digested with 10 U of *Dpn*I was incubated 37°C for 1 h. Reactions were directly transformed into chemically-competent XL-1. P*phoP*pJET plasmid DNA was prepared from overnight cultures of transformants using the PureYield Plasmid Miniprep System (Promega) as per manufacturer’s instructions. Mutagenesis of the OmpR-I binding site was confirmed by DNA sequencing of the *phoP* promoter.

1. Datsenko KA & Wanner BL (2000) One-step inactivation of chromosomal genes in *Escherichia* *coli* K-12 using PCR products. Proc Natl Acad Sci USA 97: 6640-6645.
2. Uzzau S, Figueroa-Bossi N, Rubino S, & Bossi L (2001) Epitope tagging of chromosomal genes in *Salmonella*. Proc Natl Acad Sci USA 98: 15264-15269.
